# Immunoglobulin Genomics in the Guinea Pig (*Cavia porcellus*)

**DOI:** 10.1371/journal.pone.0039298

**Published:** 2012-06-22

**Authors:** Yongchen Guo, Yonghua Bao, Qingwen Meng, Xiaoxiang Hu, Qingyong Meng, Liming Ren, Ning Li, Yaofeng Zhao

**Affiliations:** 1 State Key Laboratory of AgroBiotechnology, College of Biological Sciences, China Agricultural University, Beijing, People's Republic of China; 2 Department of Basic Immunology, Xinxiang Medical University, Xinxiang, People's Republic of China; 3 National Key Laboratory of Veterinary Biotechnology, Harbin Veterinary Research Institute, Chinese Academy of Agricultural Sciences, Harbin, People's Republic of China; 4 College of Animal Science and Technology, Qingdao Agricultural University, Qingdao, People's Republic of China; National Institute on Aging, United States of America

## Abstract

In science, the guinea pig is known as one of the gold standards for modeling human disease. It is especially important as a molecular and cellular biology model for studying the human immune system, as its immunological genes are more similar to human genes than are those of mice. The utility of the guinea pig as a model organism can be further enhanced by further characterization of the genes encoding components of the immune system. Here, we report the genomic organization of the guinea pig immunoglobulin (Ig) heavy and light chain genes. The guinea pig IgH locus is located in genomic scaffolds 54 and 75, and spans approximately 6,480 kb. 507 V_H_ segments (94 potentially functional genes and 413 pseudogenes), 41 D_H_ segments, six J_H_ segments, four constant region genes (μ, γ, ε, and α), and one reverse δ remnant fragment were identified within the two scaffolds. Many V_H_ pseudogenes were found within the guinea pig, and likely constituted a potential donor pool for gene conversion during evolution. The Igκ locus mapped to a 4,029 kb region of scaffold 37 and 24 is composed of 349 V_κ_ (111 potentially functional genes and 238 pseudogenes), three J_κ_ and one C_κ_ genes. The Igλ locus spans 1,642 kb in scaffold 4 and consists of 142 V_λ_ (58 potentially functional genes and 84 pseudogenes) and 11 J_λ_ -C_λ_ clusters. Phylogenetic analysis suggested the guinea pig’s large germline V_H_ gene segments appear to form limited gene families. Therefore, this species may generate antibody diversity via a gene conversion-like mechanism associated with its pseudogene reserves.

## Introduction

The guinea pig (*Cavia porcellus*), also called the cavy, is a species of rodent belonging to the family *Caviidae*. This animal has been used in scientific experimentation since the 17th century. During the 19th and early 20th centuries, the guinea pig was a popular experimental animal for studying prevalent bacterial diseases such as tuberculosis and diphtheria [1], resulting in the epithet “guinea pig" being used to describe a test subject. Guinea pigs are currently still used in research, primarily as models for human diseases, including juvenile diabetes, tuberculosis, scurvy, and pregnancy complications [2], [3], [4], [5], [6], [7], [8].

Immunoglobulins (Igs) are only expressed by jawed vertebrates [9], [10], [11] and are usually composed of two identical heavy (H) chains and two identical light (L) chains. Exceptions include shark IgNAR and camelid IgGs, which are only comprised of heavy chains [9], [10], [11]. To date, mammalian Ig genes are organized into a ‘translocon’ configuration [12]. In the heavy chain locus, multiple variable (V_H_), diversity (D_H_), and joining (J_H_) gene segments are followed by μ, δ, γ, ε, and α genes [13]. In the kappa encoding locus, multiple joining (J_κ_) region gene segments are present within a cluster, followed by a single constant (C_κ_) gene, whereas in the lambda encoding locus, joining (J_λ_) and constant (C_λ_) genes occur as J_λ_-C_λ_ blocks, which usually have multiple copies [14].

The word “guinea pig" is synonymous with scientific experimentation, but little is known about its Ig genes. We therefore used the recently available genome data of guinea pig provide as an opportunity to study the Ig genes of this species. Our study aimed to characterize the guinea pig IgH and IgL loci, in an effort to promote a better understanding of the immune system and evolutionary divergence of the Ig genes in placental mammals.

## Materials and Methods

### Guinea Pig Genome Sequence

Guinea pig (*C. porcellus*) genome data were obtained from the Ensembl database (http://www.ensembl.org), and the Broad Institute conducted genome sequencing and assembly (cavPor3, 6.79× coverage, Jul 2008). High-coverage ensured increased accuracy of the genome analysis results.

**Figure 1:**
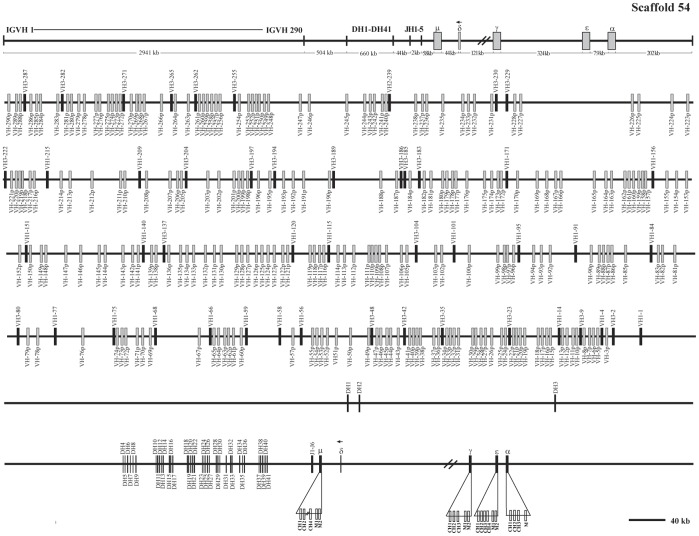
The guinea pig IgH locus in scaffold 54. The guinea pig IgH locus length is approximately 4,302 kb from the most 5′ V_H_ segment (V_H_-290p) to the most 3′ end of α gene in genomic scaffold 54. Filled bars: potentially functional V_H_ genes; open bars: V_H_ pseudogenes and V-p; D_H_: diversity genes; J_H_: joining genes; μ: IgM coding gene; δ: IgD fragment; γ: IgG coding gene; ε: IgE coding gene and α: IgA coding gene. V_H_ and D_H_ genes are numbered based on the families and their order. The number before the dash (V_H_n) indicates the family, while the number after dash represents the genomic position. J_H_ genes are numbered based on the order of their locations in the locus. Double slashes indicate gaps >10 kb. The unidirectional arrowheads above δ gene segments in scaffold 54 indicate that its direction is opposite to the other heavy chain constant genes transcriptional direction. The scale does not apply the upper frame diagram.

### Identification of the Guinea Pig Ig Genes

Guinea pig Ig constant region genes were retrieved on the basis of comparing guinea pig and human Ig gene sequences (http://genome.ucsc.edu/). FUZZNUC, an online software (http://mobyle.pasteur.fr/cgi-bin/portal.py?#forms::fuzznuc), was used to find adjacent recombination signal sequences (RSSs) for the identification of variable, diversity, and joining gene segments. Five or more mismatched bases were allowed to cover all genes.

### Total Ribonucleic Acid (RNA) Extract and 3′ RACE

Total RNA was extracted from spleen tissues of three male HARTLY guinea pigs using TRIzol Reagent (Invitrogen, USA) and pooled equally. Animals were treated in accordance with the China Agricultural University on the protection of animals used for experimental and other scientific purposes. The study was approved by animal welfare committee of China Agricultural University with approval number XK257.

Complementary deoxyribonucleic acid (cDNA) was synthesized from 1 µg of total RNA using 3′RACE-reverse transcription primers. The first polymerase chain reaction (PCR) amplification of each Ig heavy chain constant region gene (μ, γ, ε, and α) was performed using the corresponding sense primer and 3′RACE-antisense primer 1. While the second PCR amplification was conducted using the corresponding sense primer and 3′RACE-antisense primer 2. All primers are displayed in Table S1.

**Figure 2:**
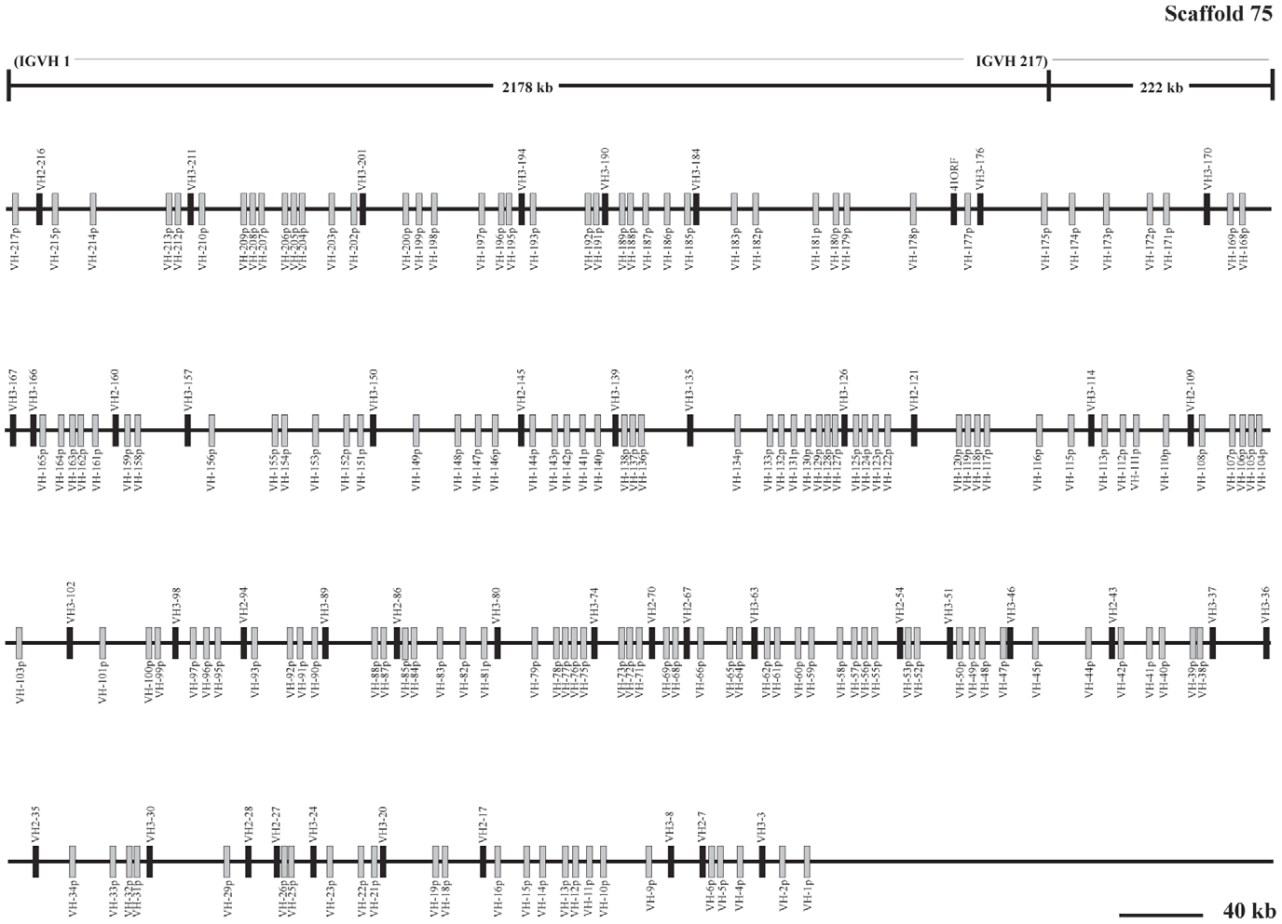
The guinea pig IgH locus in scaffold 75. The guinea pig IgH locus length is approximately 2,177 kb from the most V_H_ (V_H_-217p) to the most end (V_H_-1p) in scaffold 75. Filled bars: potentially functional V_H_ genes; open bars: V_H_ pseudogenes. The scale does not apply the upper frame diagram.

### Southern Blotting Analysis of Genomic DNA Sample

Guinea pig liver genomic DNA was isolated according to the method as previously described [15]. The V_H_ gene family-specific probes belonging to guinea pig V_H_1, V_H_2 and V_H_3 gene families were labeled using a PCR digoxygenin probe synthesis kit (Roche, Germany) with primers designed from V_H_1-95, V_H_2-17 and V_H_3-157 (The primer sequences are displayed in Table S1). Ten micrograms of genomic DNA digested separately with *Bam*H I, *Ec*oR I, *Hin*d III and *Xba* I (New England Biolabs, USA) were loaded into each well of a 0.8% agarose gel and electrophoresed for 12 h, transferred to a positively charged nylon membrane (Roche, Germany). Hybridization and detection were conducted following the manufacturer’s instructions.

### Sequence and Phylogenetic Analysis

Multiple sequence alignments were edited and handled with the Megalign software program [16], and the Clustal W and Clustal X algorithms [17], [18], before being analysed using BioEdit [19]. Comparative phylogenetic trees were constructed using the PHYLIP 3.67 [20] software and TreeView [21] based on the final nucleotide alignment. The neighbor-joining algorithm was used for phylogenetic analysis and bootstrap support was provided by 1000 replicates. Sequences from other species used in our phylogenetic analyses and sequence alignments are presented in Figures S1, S2, and S3 and Table S2.

### Dot Matrix Analysis

Pairwise dot matrix comparisons were made using DNAMAN software (window size  = 30-bp, mismatch limit  = 9-bp) to identify potential alignment of nucleotide bases between the sequences.

**Figure 3:**
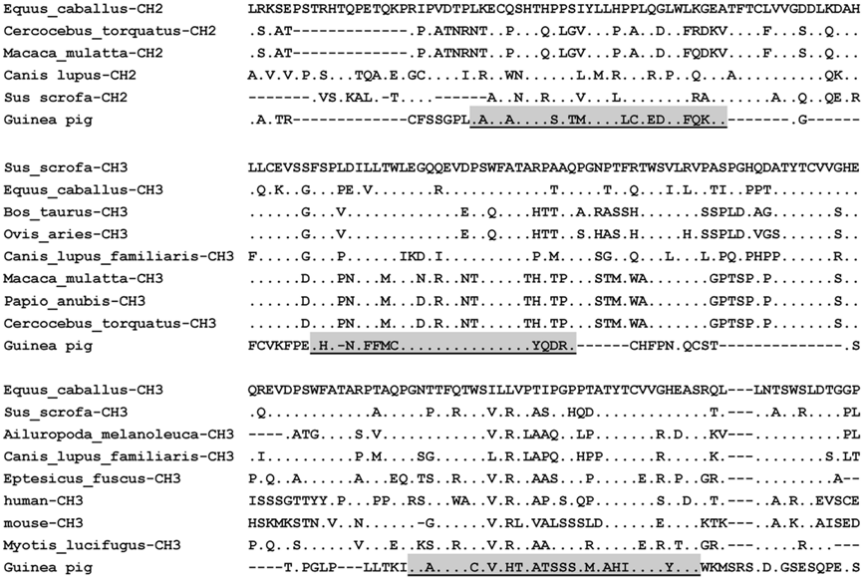
Alignment of the guinea pig IgD remnants with other mammalian IgD domains. Amino acid residues that are identical to the top counterpart in every panel are shown as dots; Gaps and missing data are indicated by hyphens.

### Definition of the V_H_/V_L_ Gene Families

In mammals, germline V_H_ and V_L_ genes are categorized into different families according to their amino acid or nucleotide sequences similarity [22]. Sequences with greater than 75% similarity are general considered to belong to the same family, while those with less than 70% similarity are placed in different gene families, and those possessing between 70% and 75% similarity are inspected on a case-by-case basis [23]. We placed potentially functional V_H_ and V_L_ gene segments sharing more than 70% similarity into the same family.

**Figure 4:**
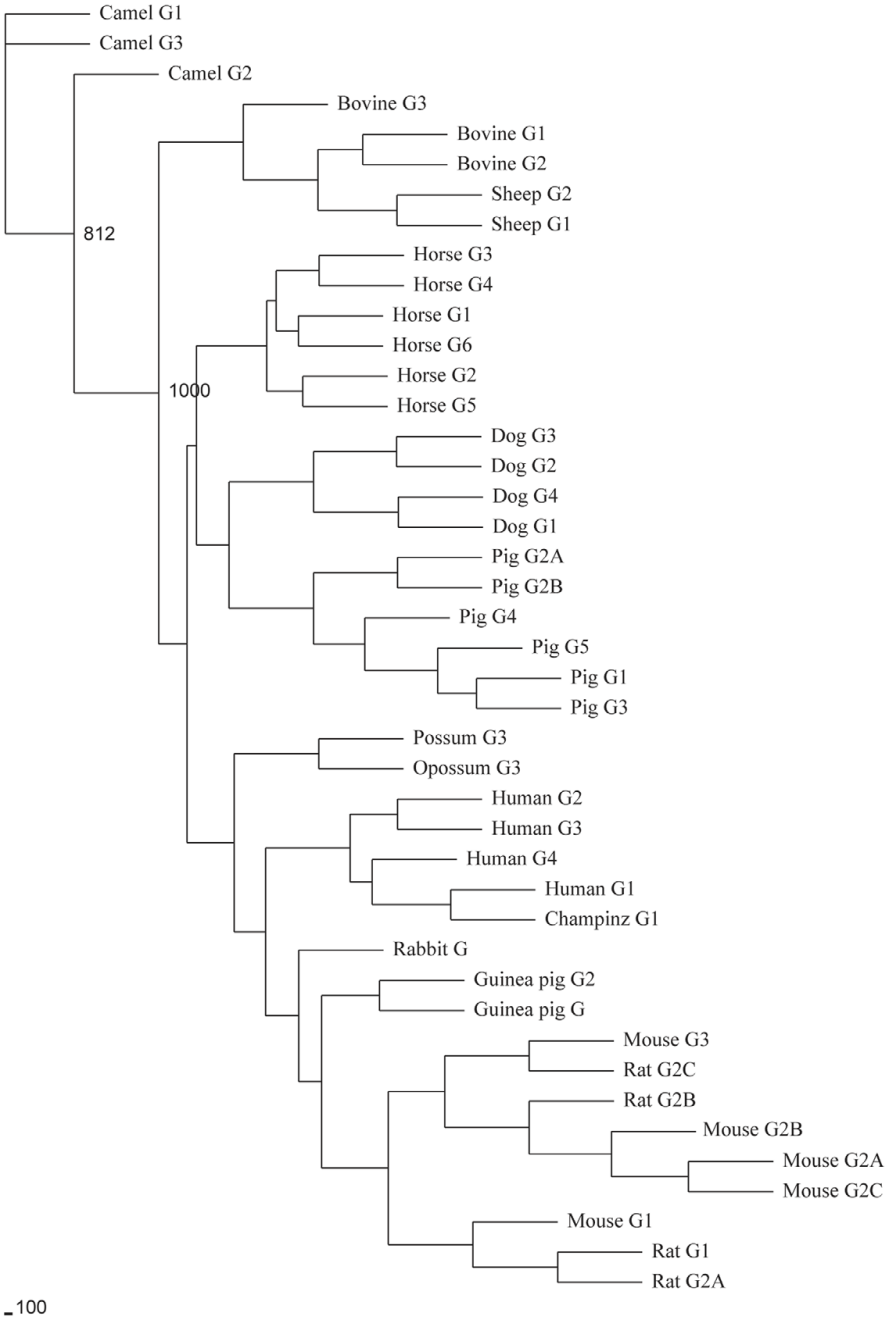
Phylogenetic analysis of mammalian immunoglobulin gamma genes. The phylogenetic tree was constructed from the amino acid sequences of the C_H_2 and C_H_3 exons with various mammalian species immunoglobulin gamma gene. The guinea pig IgG genes were clustered with rodents IgG genes.

## Results

### Guinea Pig IgH Locus

Analysis of the genomic sequence revealed that the guinea pig IgH locus is located within genomic scaffolds 54 and 75 (Figure 1, Figure 2). The entire IgH locus spans approximately 6,480 kb of the two scaffolds (4,302 kb in scaffold 54 and 2,178 kb in scaffold 75). The total length is an estimate due to the existence of sequence gaps (Figure 1, Figure 2). Six J_H_ segments, 507 V_H_, 41 D_H_, four constant region genes (μ, γ, ε and α) and a reverse δ trace (marked with an arrow towards the left) were identified within the two scaffolds. Locations of the annotated IgH genes on the guinea pig genome are displayed in Table S3.

**Figure 5:**
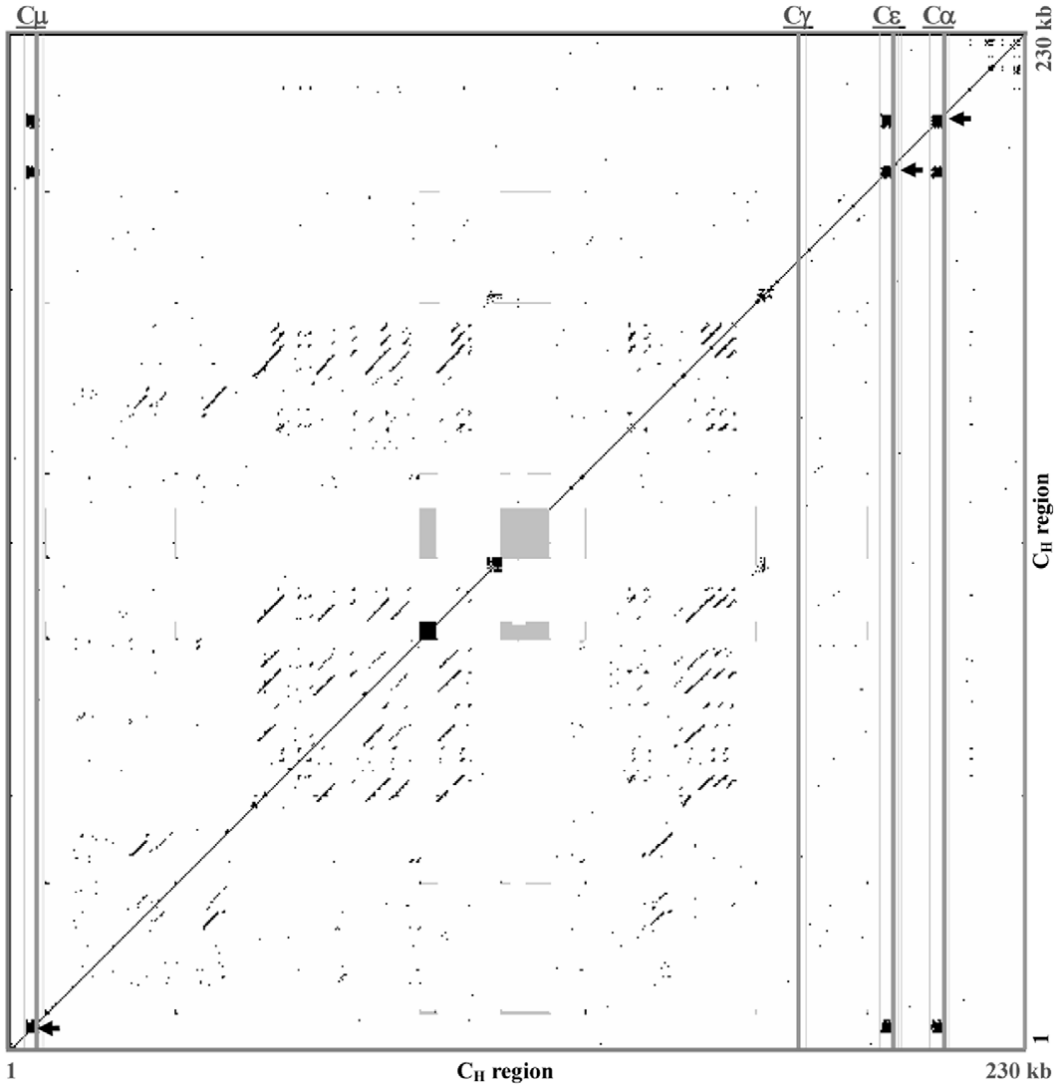
Dot plot comparison of the guinea pig C_H_ (μ, ε, γ and α) region. A dot matrix representing repetitive sequences of guinea pig C_H_ (μ, ε, γ and α) genes. Switch regions are indicated by black-squared boxes and marked with arrowheads, and gaps are indicated by grey-squared boxes. Positions of C_H_ genes are indicated as grey vertical lines. The dots represent homologies with a search length of 30 bp and maximum of 9 bp mismatches.

**Figure 6:**
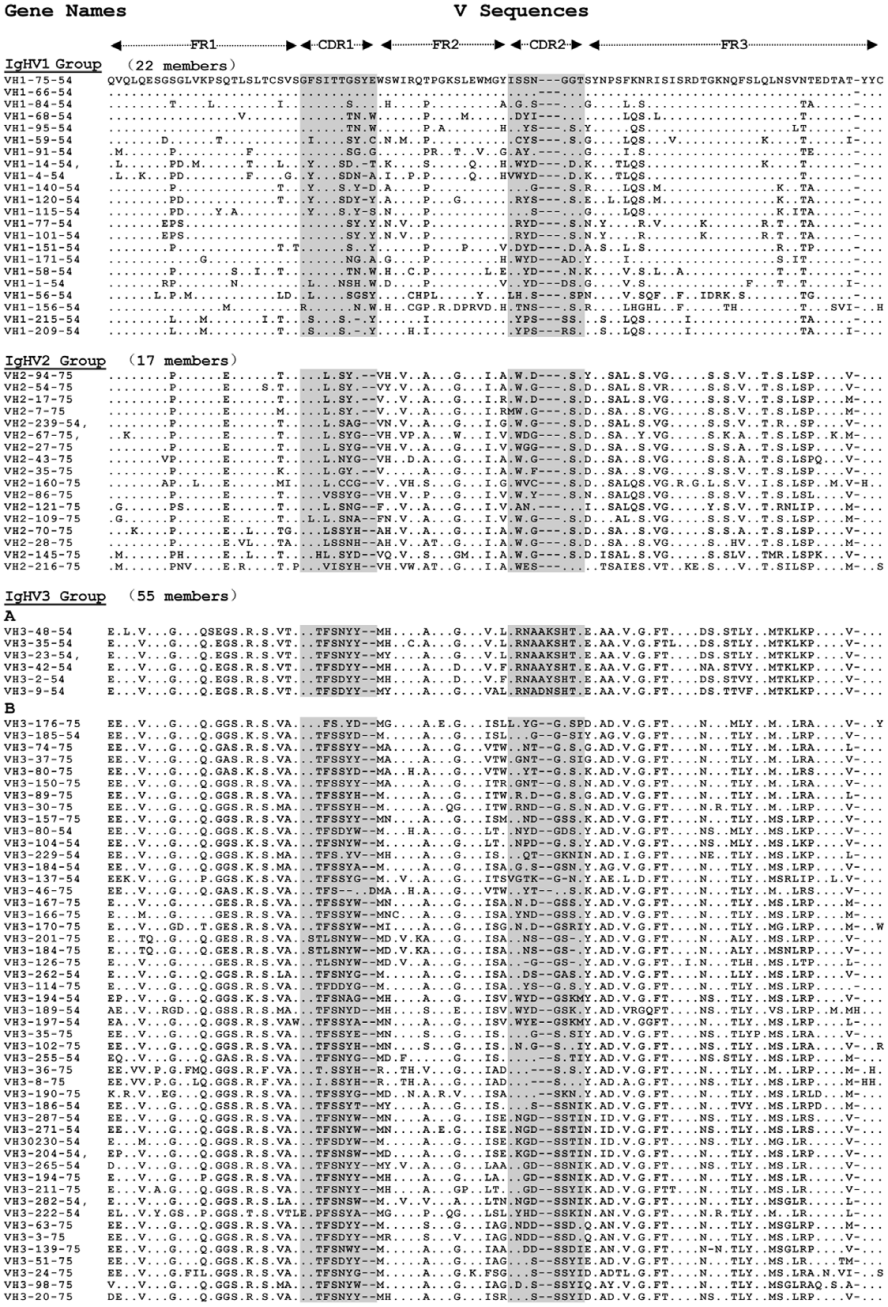
The alignment of deduced amino acid sequences of guinea pig 94 functional V_H_ genes. The sequences of functional V genes were analyzed by Megalign software and BioEdit programe. The Designation of framework regions (FR) and complementarity determining regions (CDR) referred to IMGT numbering system, and the CDR region are indicated by grey-squared background. amino acid sequences that are the same as the top segment, V_H_1-75-54, are indicated with dots. Dashes mean gaps introduced to make the alignment.

**Figure 7:**
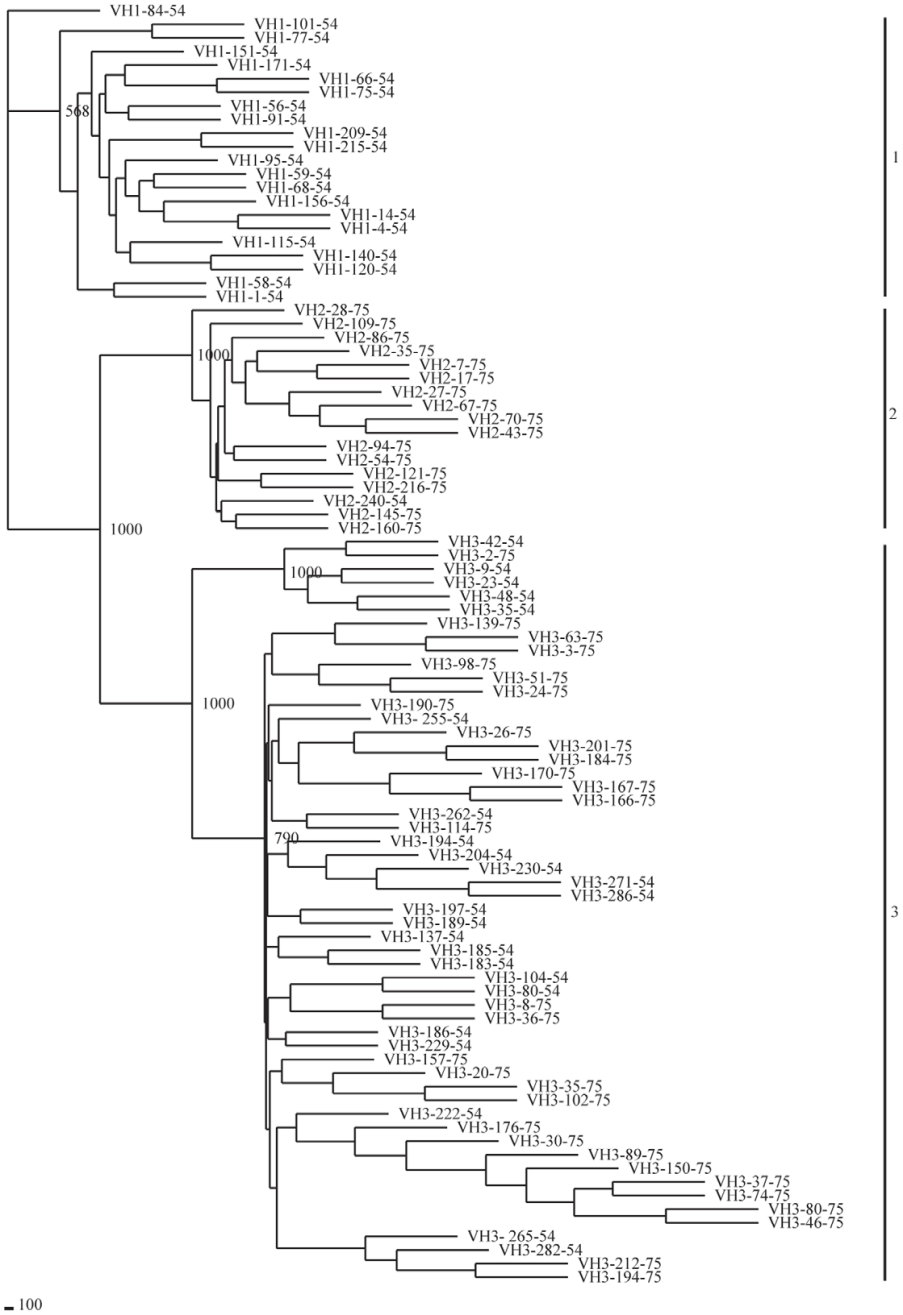
Phylogenetic analysis of 94 guinea pig V_H_ genes. A phylogenetic tree of nucleotide sequences of 94 guinea pig potentially functional V_H_ segments was constructed. The three identified V_H_ gene families are labeled with Arabic numerals.

**Figure 8:**
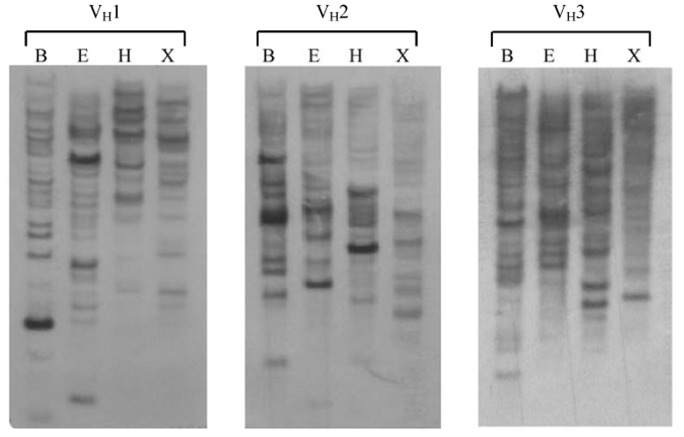
Southern blotting analysis of guinea pig genomic DNA. Southern blotting analysis of the guinea pig heavy chain variable region genes. Genomic DNA was digested with *Bam*H I (B), *Ec*oR I (E), *Hin*d III (H) and *Xba* I (X), and hybridized with probes for each of three guinea pig families.

### Analysis of the Guinea Pig Constant Region Genes

To acquire the cDNA sequence for four heavy chain isotypes (μ, γ, ε, and α), 3′RACE were performed on splenic RNA using specific primers. Using this strategy we successfully identified genes encoding the constant region exons for IgM, IgG, IgE, and IgA (Figure S4). As predicted from the analysis of the guinea pig Ig genomic genes, the domain structure for the four isotypes was typical of that for other mammalian species (Figure S5).

Within the guinea pig genome, C_H_3 and parts of the C_H_4 segments of the μ gene were determined to be missing because of the existence of gaps (Figure 1). By using the 3′ RACE method, the complete secreted IgM, including four exons, was successfully cloned (Figure S4–S5).

Most mammals also express the δ gene, with the exception of the rabbit [24] and opossum [25]. The area predicted to contain an IgD C region, and in particular the 3′ region of the IgM exons between IgM and IgG, as well as the whole cavPor3 assembly, was thoroughly searched in two different orientations for coding sequences that might correspond to a putative IgD. These searches detected δ trace fragments that are homologous with mammalian IgD (Figure 3) and showed an opposite transcription direction to the upstream μ gene. Based on sequence alignment, the three-fragment δ trace was found to belong to the C_H_2 and C_H_3 domains (Figure 3).

**Figure 9:**
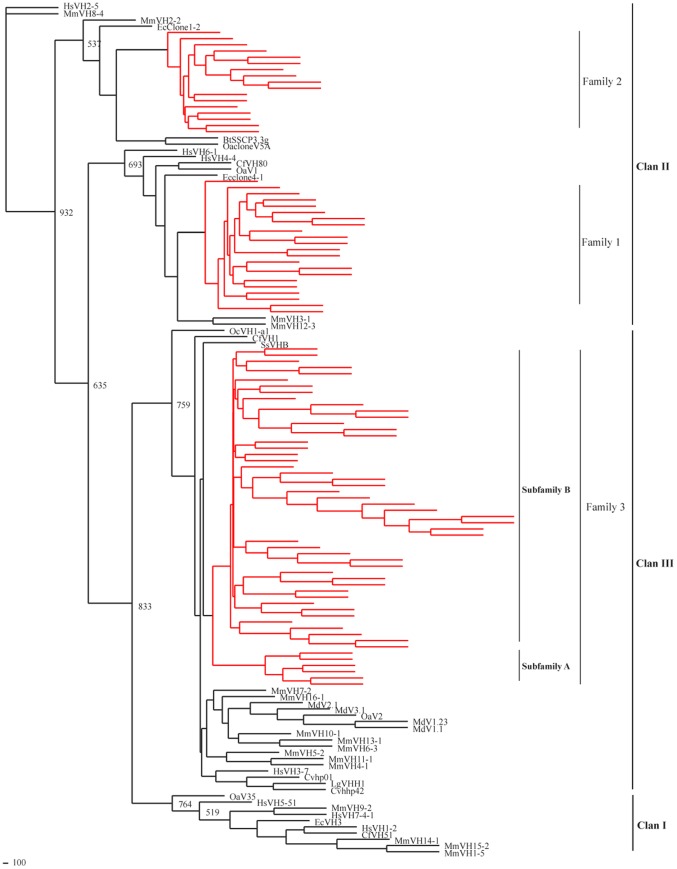
Phylogenetic analysis of mammalian V_H_ genes. Guinea pig sequences are represented by red branches, other species sequences are represented by black branches. Three V_H_ families of guinea pig were clustered respectively with other mammalian V_H_ families, and the V_H_ families of guinea pig belong to ClanII and ClanIII. The V_H_3 family could be divided into two subfamilies.

Although two IgG isotypes (IgG1 and IgG2) were previously identified in domestic guinea pig serum [26], we only identified one IgG gene in the guinea pig genome perhaps due to sequence gaps. Our sequence alignment further showed that the recognized IgG in guinea pig genome shared the highest similarity with IgG1 (six amino acid difference) (Figure S6). Interestingly, we were just able to clone the IgG2 mRNA transcript by 3′ RACE. To address this question, a further Southern blotting experiment using the C_H_1 exon (high similarity between IgG1 and IgG2) as a probe, which showed that there were more than one γ genes in the guinea pig genome (Figure S7). Taken together, these data suggested that the guinea pig had two γ genes in its genome but only one was preferentially expressed.

**Figure 10:**
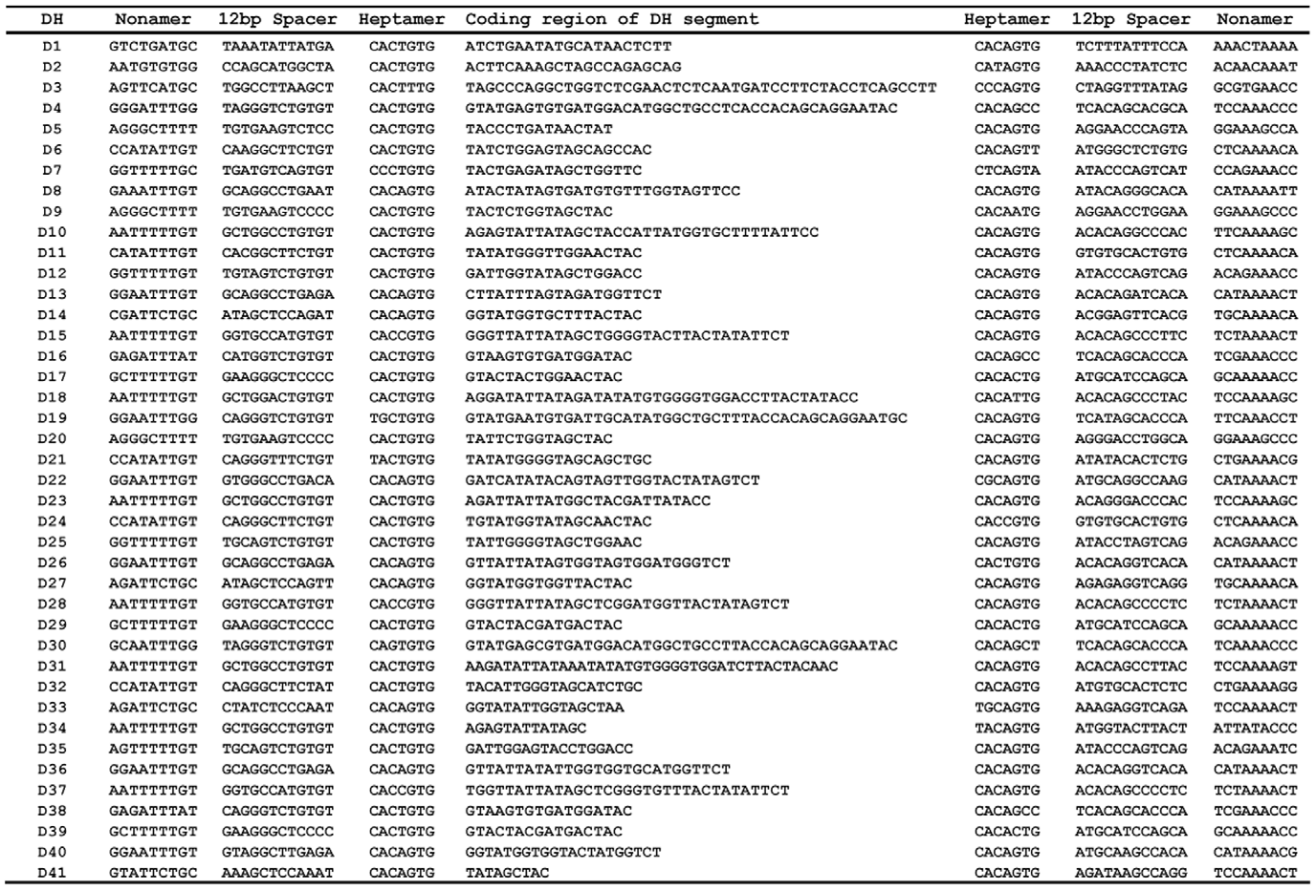
The nucleotide sequences of D_H_ genes. D_H_ coding region nucleotide sequences are represented together the RSS elements (nonamer and heptamer sequences).

**Figure 11:**
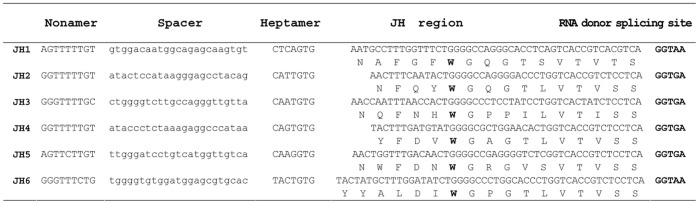
The nucleotide sequences of J_H_ genes. J_H_ nucleotide sequences are represented together the RSS elements (nonamer and heptamer sequences) and RNA donor splicing site.

**Figure 12:**
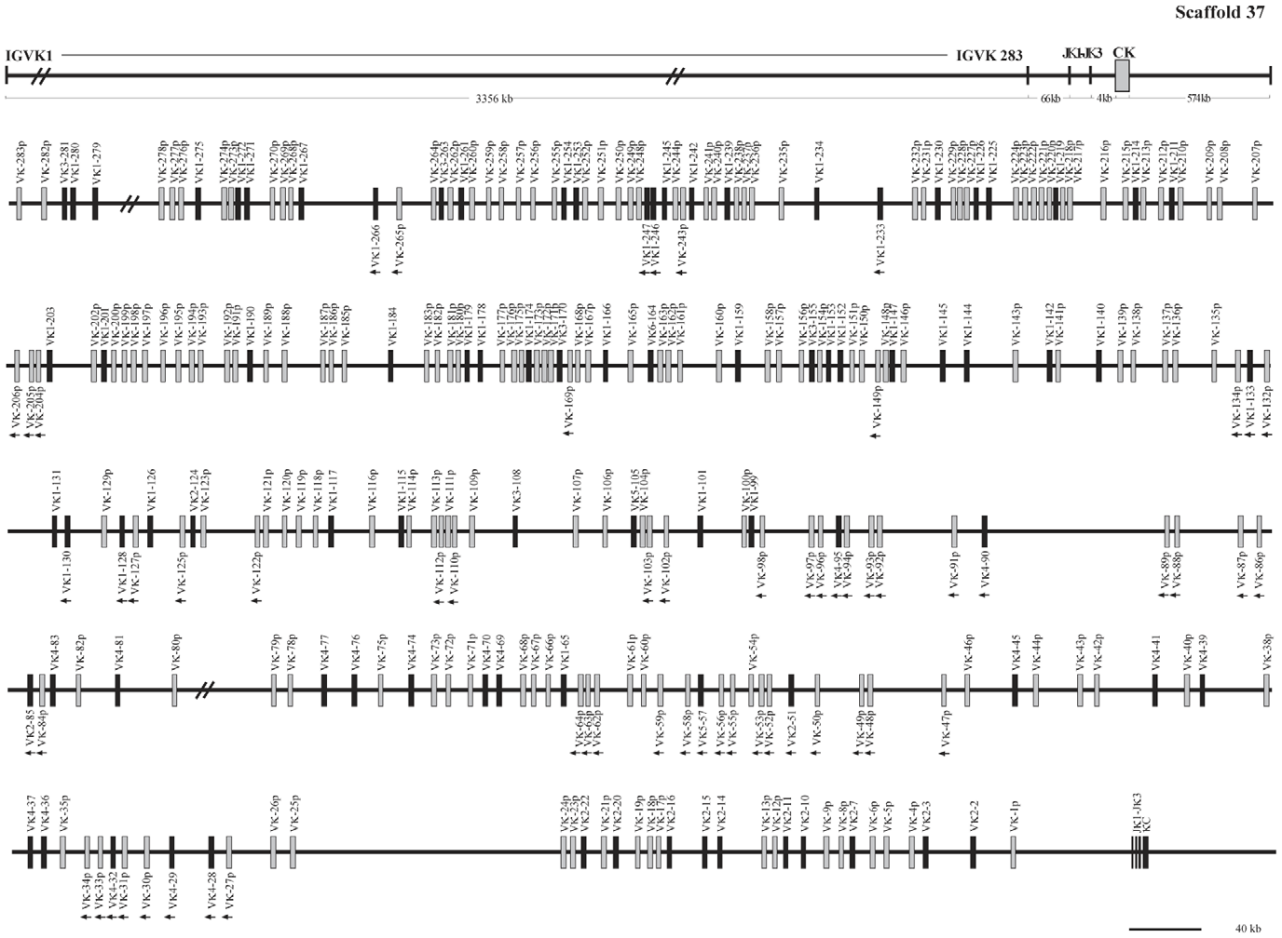
The guinea pig Igκ locus in scaffold 37. The guinea pig Igκ locus is in scaffold 37, the potentially functional V_κ_ gene segments are shown in filled bars, and pseudogenes are represented by open bars. The unidirectional arrowheads below V_κ_ gene segments on scaffold 37 indicate that their transcriptional direction is opposite to downstream J_κ_ segments. Double slashes indicate gaps >10 kb. The scale does not apply the upper frame diagram.

**Figure 13:**
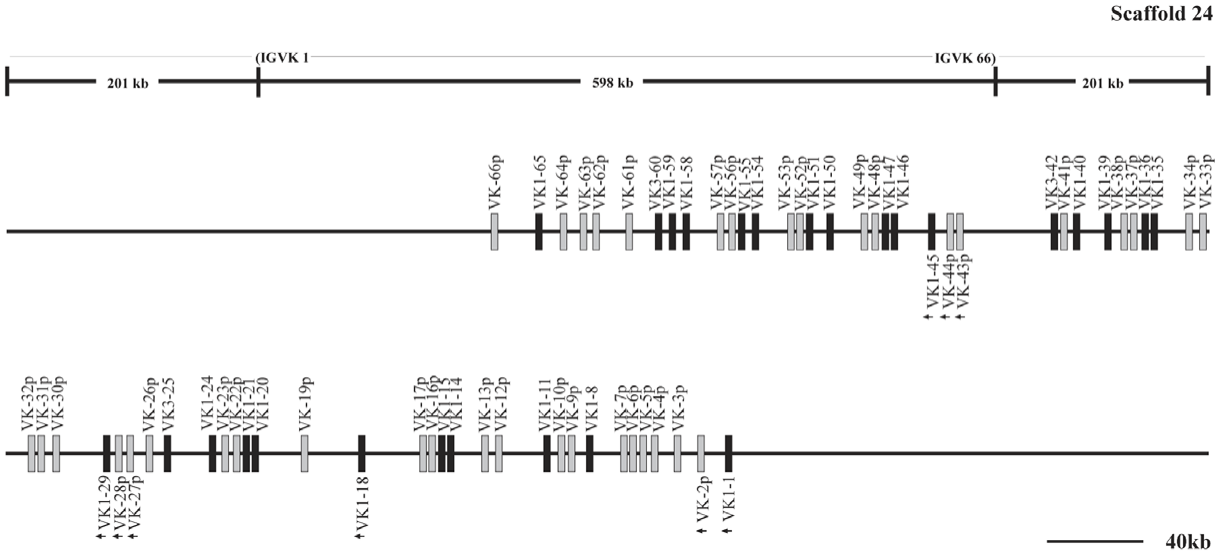
The guinea pig Igκ locus in scaffold 24. The guinea pig Igκ locus is in scaffold 24, the potentially functional V_κ_ gene segments are shown in filled bars, and pseudogenes are represented by open bars. The unidirectional arrowheads below V_κ_ gene segments merely indicate a transcriptional direction different from that of the remaining V_κ_ gene segments in scaffold 24. The scale does not apply the upper frame diagram.

**Figure 14:**
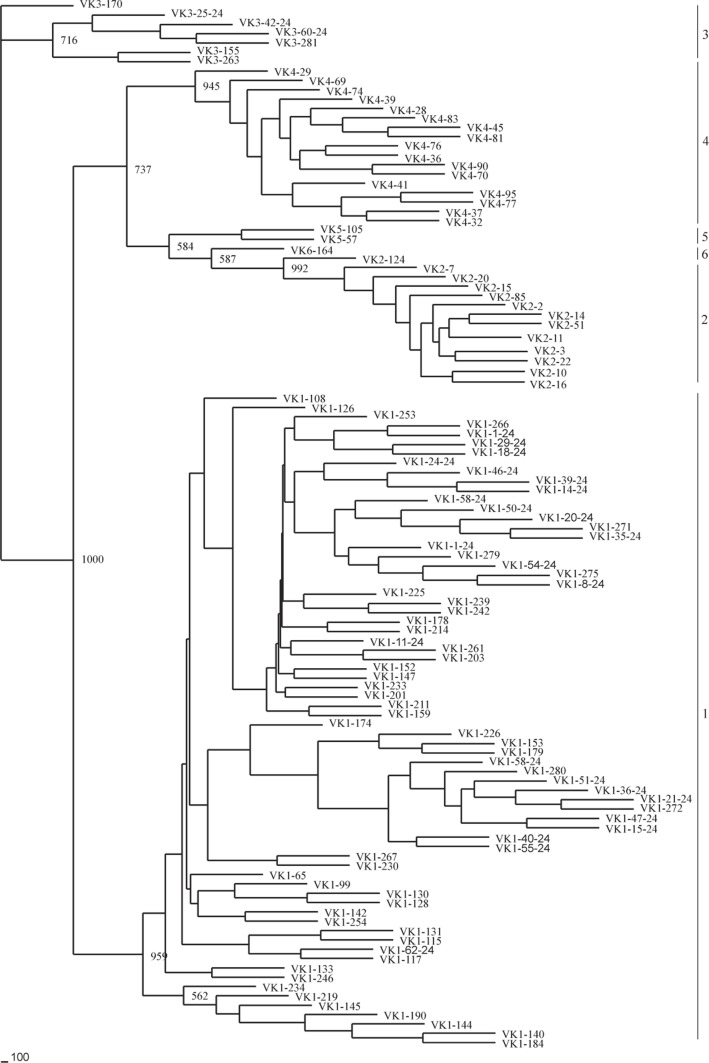
Phylogenetic analysis of the 111 guinea pig Vκ genes. A phylogenetic tree of the nucleotide sequences of 111 guinea pig V_κ_ segments were constructed. The six V_κ_ gene families are labeled with Arabic numerals.

**Figure 15:**
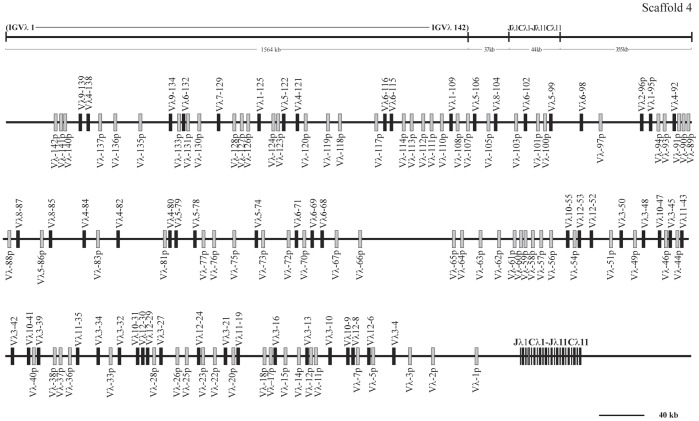
The guinea pig Igλ locus. The guinea pig Igλ locus is in scaffold 4, the potentially functional V_λ_ gene segments are shown in filled bars, and pseudogenes are represented by open bars.

**Figure 16:**
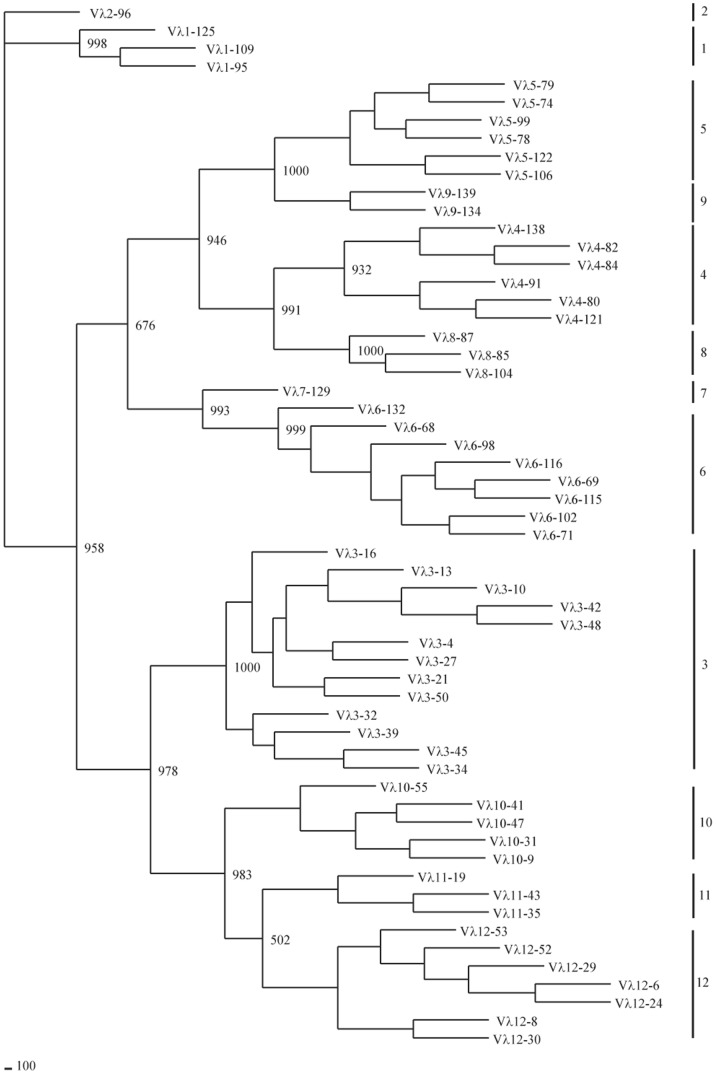
Phylogenetic tree analysis of the 58 guinea pig V_λ_ genes. A phylogenetic tree of the nucleotide sequences of 58 guinea pig V_λ_ segments were constructed. The eleven V_λ_ gene families are labeled with Arabic numerals.

**Figure 17:**
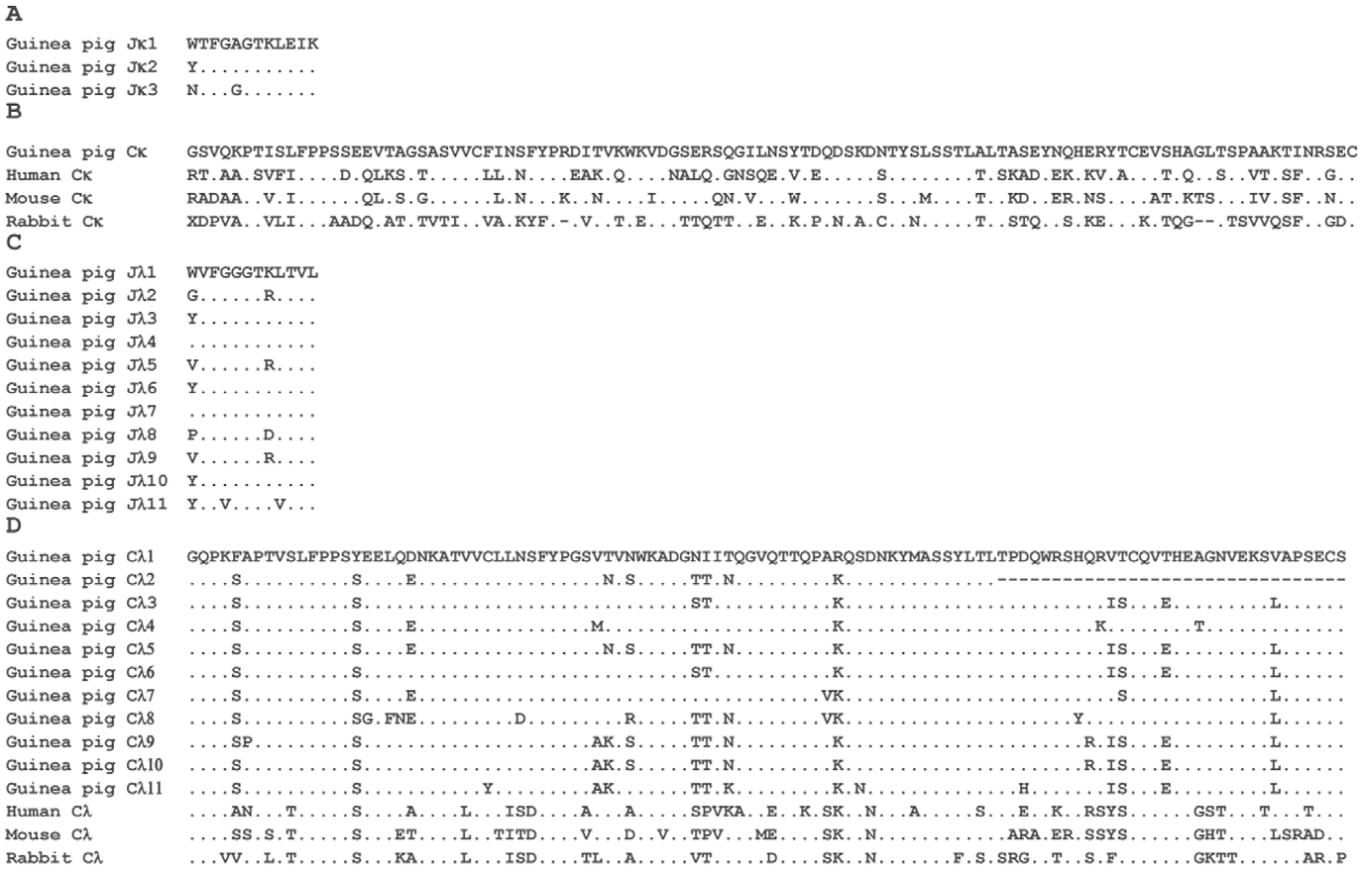
The alignment of amino acid sequences of J and C genes from guinea pig IgL chains. A, Alignment of the deduced amino acid sequences of the three guinea pig J_κ_ gene segments. B, Alignment of the amino acid sequences of the C_κ_ proteins from human, mouse and rabbit. C, Alignment of the deduced amino acid sequences of the eleven guinea pig J_λ_ gene segments. D, Alignment of the amino acid sequences of the C_λ_ proteins from human, mouse and rabbit. Amino acid residues that are identical to the top counterpart in every panel are shown as dots; Gaps and missing data are indicated by hyphens.

A phylogenetic tree constructed with C_H_2 and C_H_3 exons of IgG from different mammalian species revealed that the guinea pig IgG genes were clustered with rodents IgG genes (Figure 4). IgG2 and IgA also exhibit a hinge region, which is thought to have evolved by condensation of the C_H_2 exon in an ancestral isotype such as IgY in birds [25], [27]. The hinge region of IgA is encoded by the 5′ end of the C_H_2 exon, as observed in other eutherian mammals [28], [29], [30] (Figure S4–S5).

In mammals, especially in humans and mice, a pentameric tandem repeat sequence is found upstream of the heavy chain constant region, which acts as a switch or S region. S regions have previously been mapped and sequenced, and are relevant in Ig class switch recombination. Such characteristic tandem repeats were also found within the upstream Cμ, Cε and Cα gene sequences of the guinea pig. Three putative S regions span 2.2 kb to 3 kb, and exhibit similar repeats (GAGCT and GGGCT) to those observed in humans and mice. However, the characteristic sequence of the switch regions could not be identified within the γ gene, most likely due to sequence gaps. Dot matrix analysis of the guinea pig S region revealed substantial nucleotide similarity with those of humans and mice (Figure 5).

### Analysis of the Guinea Pig V_H_ Gene Segments

A total of 507 V_H_ segments were identified in scaffolds 54 and 75 (Figure 1, Figure 2). Ninety-four of these appeared to be potentially functional because they contained leader exons (L), uninterrupted open reading frames (ORF), downstream RSS, and a V gene domain (framework regions and complementarity determining regions). The remaining 413 segments that contain either in-frame stop codons or are partial sequences were designated as pseudogenes (Figure 1, Figure 2 and Table S3). Given that gaps existed within the assembly, it is possible that as yet unidentified V_H_ genes are also present in the guinea pig genome.

Phylogenetic analysis and multiple sequence alignments, including all functional guinea pig germline V_H_ gene segments, revealed the V_H_ gene families 1, 2 and 3, which were comprised of 22, 17 and 55 members, respectively (Figure 6, Figure 7). The largest family, V_H_3, could be further divided into two subfamilies (Figure 6, Figure 7). We also performed Southern blotting to verify the multiple numbers of V_H_ genes of different families in the guinea pig genome (Figure 8).

We chose all potentially functional guinea pig V_H_ sequences of and V_H_ sequences that represented previously reported gene families from other mammalian species to construct a neighbor-joining phylogenetic tree [31], [32] (Figure 9). The phylogenetic tree indicated that the mammalian V_H_ gene families were classified into three clans [33]. The guinea pig V_H_ families 1 and 2 belonged to clan II, and family 3 belonged to clan III.

### Analysis of the Guinea Pig D_H_ and J_H_ Gene Segments

Approximately 504 kb downstream from the last V_H_ segment (V_H_1-1), we identified 41 D_H_ segments (i.e., D_H_1–D_H_41). They spanned a 660 kb region of DNA in scaffold 54 (Figure 1). Each D_H_ segment was flanked by conserved RSS elements composed of heptamers and nonamers separated by 12 bp spacers and existed within at last one alternative reading frame (Figure 10 and Figure S8), suggesting that they are potentially functional.

J_H_ region contained six genes (designated J_H_1 to J_H_6) spanning approximately 2 kb. Each J_H_ gene had an upstream RSS element with a 22–23 bp spacer, ORF, and a downstream RNA donor-splicing site at the 3′ end, suggesting that they are potentially functional (Figure 11).

### Guinea Pig Igκ Locus

The guinea pig Igκ chain is located in scaffolds 37 and 24, and spans an approximately 4,029 kb genomic region (Figure 12, Figure 13 and Table S4). A total of 349 V_κ_ genes were identified. Further analysis revealed that 111 V_κ_ genes might be potentially functional genes, given that they contained an L sequence, ORF, RSS and V domain. The remaining 238 segments contain either in-frame stop codons or frameshifts, and are thus designated as pseudogenes. Based on sequence analysis, the 111 potentially functional V_κ_ genes were divided into six families (V_κ_1–V_κ_6), which contained 69, 15, 7, 17, 2, and 1 member/s, respectively (Figure 14). In addition, 222 V_κ_ genes were arranged in the same transcriptional orientation and exhibited downstream J_κ_ and C_κ_, and 61 V_κ_ segments and a reverse transcriptional direction in scaffold 37. Downstream of the V_κ_ genes, three J_κ_ gene segments were identified that spanned 0.6 kb. Furthermore, approximately 4 kb downstream from the last J_κ_, a single C_κ_ gene was identified.

### Guinea Pig Igλ Locus

Guinea pig Igλ chain genes were identified in scaffold 4, and spanned an approximately 1,642 kb length (Figure 15, Table S5). Of 142 germline V_λ_ genes identified, 58 segments were categorized as potentially functional genes, and the remaining 84 were differentiated as pseudogenes. Based on sequence similarity analysis, the potentially functional guinea pig V_λ_ genes were assigned to twelve families (Figure 16), comprised of 3, 1, 13, 6, 6, 8, 1, 3, 2, 5, 3 and 7 member/s, respectively. In contrast to the V_κ_ genes, all the V_λ_ genes have the same transcriptional orientation as the downstream J_κ_ and C_κ_ regions. At the 3′ end of this scaffold, 11 J segments and C segments were organized in tandem and spanned 44 kb, while the C_λ_2 exhibited less structural integrity owing to the presence of gaps. The sequence alignments of J_λ_ and C_λ_ gene segments are shown in Figure 17.

## Discussion

Rodents are a ubiquitous group of species worldwide, representing nearly half of all mammalian species, which evolved from a common ancestor shared with the lagomorphs approximately 62**–**100 million years ago [34], [35]. The classification of the guinea pig within the family Caviidae and genus Cavia is somewhat controversial because the origin of this rodent is poorly known, with current classification data mostly relying on fossils or genetic relationships [36], [37], [38], [39], [40], [41], [42], [43], [44]. We therefore analyzed the Ig genes sequences of the guinea pig, not only to better understand the immune system of this species, but also to provide data for comparative studies of mammalian Ig genes.

The IgH locus of mammals is arranged in a “translocon" configuration [32], [45], [46], [47], [48], [49], [50], [51], [52]. In the present study, we characterized the guinea pig Ig genes based on recently released genomic data and our experimental results. The guinea pig IgH locus in a configuration of V_H_ (507)-D_H_ (41)-J_H_ (6)-Cμ-ψδ-Cγ2-Cε-Cα spanning at least 6,480 kb in two scaffolds may be largest in all mammalian species studied so far.

On the basis of sequence analysis, we identified a single μ, γ, ε and α gene within the guinea pig genome. We also found three fragments of δ gene in an opposite direction downstream from the μ gene. Due to sequence gaps, it is not certain if an additional functional δ gene also exists in this species. We have also tried to confirm the sequence and orientation of the δ gene fragments by genomic PCR to eliminate assembly error. The sequences of the δ gene fragments were verified. Because two genomic fragments (approximately two kb and six kb) between μ and δ gene can not be successfully amplified, so the orientation of the δ gene fragments remains a question.

Many placental mammals, such as human, cow, sheep, horse and dog, have a single functional δ gene. Except for a functional δ gene which consists of ten C_H_ domains, a reverse δ pseudogene was previously observed in the platypus IgH locus [53], while in the elephant genome, only one Cδ3 remnant fragment was identified [51]. In camelids, Cδ3 exon appears to be highly mutated [54]. In the rabbit [24] and opossum (marsupial) [25], the δ gene has clearly been shown to be missing in their genomes, indicating that the δ gene might be not as essential as the μ gene in the humoral immunity.

IgG is an important antibody molecule, which is believed to have initially evolved 600 million years ago [55]. The structure of γ gene usually contains three C_H_ domains and one hinge domain. Different IgG subclasses have been reported in the majority of mammalian species, ranging from one in the rabbit [56], two in sheep [57], three in cattle [58], four in human and rat [49], [59], five in mouse [60], six in pig [61], seven in horse [62], up to nine in elephant [51]. In the guinea pig, two IgG subclasses are identified, and they share about 73% amino acid similarity in C_H_ domains. It has been postulated that different γ subclasses are derived from gene duplications of ancestral γ gene in mammals [63]. Three ancestral γ genes of mouse and rat can evolve multiple γ genes by gene duplications [64], [65], and the divergence of the γ genes of human depends on one ancestral γ gene and duplicated Cγ-Cγ-Cε-Cα fragments [66].

In different mammalian species, the number of V_H_ genes and the ratio between V_H_ functional genes and V_H_ pseudogenes vary significantly, even between closely related species, like mice and rats. For example, there are 60 pseudogenes and 44 functional V_H_ genes in humans, whereas in cows, only 6 pseudogenes and 11 functional genes have been identified [49], [67], [68]. The guinea pig germline V_H_ repertoire contains at least 507 V_H_ gene segments, which is the largest number in mammals studied to date. A large number of these germline V_H_ genes may greatly contribute to guinea pig antibody diversity. Another notable feature is the number of guinea pig V_H_ pseudogenes, which amount to 81% of the total V_H_ genes (413 pseudogenes vs. 507 total genes). The high frequency of gene duplication in variable region generated multiple V_H_ copies, many of which became pseudogenes due to genomic drift [68]. The V_H_ pseudogenes are not truly nonfunctional in some species, for example, in rabbits, the pseudogenes usually are used to generate immunoglobulin diversity by gene conversion [67].

Guinea pig V_H_ gene segments were also divided into three gene families (families 1, 2 and 3), which were orthologous to human V_H_ families 4, 2 and 3, respectively. The guinea pig exhibited a multiple gene family group, as observed in dogs (three families) [69], humans (seven families) [70], [71], horses (seven families) [32], [72] and mice (sixteen families) [73], yet different from the single family group observed in sheep [57], rabbits [74], camels [75], swine [76] and cattle [72]. The guinea pig V_H_ genes were also classified into different clans as in other mammalian species [50], [52], [67], [72], [74], [77], [78], [79]. Some reports have revealed that representative V_H_ genes belong to all three V_H_ clans in the human, mouse, cat and dog [67], [77]. This characteristic is different in cattle, horses and sheep [50], [52], [72], [78], [79], which have lost much of their ancestral repertoire, and the V_H_ genes only belong to clan II. Pigs and rabbits only have clan III genes [74], [77], [79]. V_H_ genes of guinea pig are distributed in clans II and III, and the majority of V_H_ genes (family 3) are most closely related to human V_H_3 (clan III), which has been proposed as the ancestral V_H_ gene family [78], [80]. These features are also found in swine and rabbits [74], [76].

The precise evolutionary relationship among mammalian lineages has not yet been resolved [81]. Results of the relevant study show that marsupials and monotremes were estimated to have separated from the common ancestor of present-day placental mammals more than 130 million years ago, while the major radiation of the placental mammals occurred approximately 70–120 million years ago [82], [83], [84], [85], [86]. Certain V gene families which descended from common ancestor genes are orthologues between nonplacental mammals and placental mammals. For example, platypus (*Monotreme*), dog and human share the same ancestral gene families (V_H_3 and V_H_4), While the American short-tailed opossum (*Monodelphis domestica*), swine, rabbit and human share ancestral V_H_3 family, and artiodactyl species share ancestral V_H_4 family. With an older evolutionary origin in present day mammals [86], platypus have two ancestral V_H_ gene families, while other mammals share one or two ancestral V_H_ gene families. These could be explained by an inactivation or loss of V_H_ gene members in these species during evolution [25]. For new V_H_ gene families in human or mouse, the divergence of V_H_ genes probably occurred after speciation.

The ratio of functional V_κ_ and V_λ_ is variable within mammalian species, with the germline V_κ_ genes being more abundant than V_λ_ genes in humans (40 V_κ_ genes *vs*. 30 V_λ_ genes) and mice (V_κ_ genes *vs*. V_λ_ genes over 95%) [87]. This is also the case for the guinea pig, in which V_κ_ germline genes are more dominant than V_λ_ (84 functional V_κ_ genes *vs.* 58 functional V_λ_ genes). It has been proposed that the priority of use of the light-chain gene isotypes at the protein level may be connected with the overall number of V gene segments [87]. It is possible that the κ chain preponderates over the λ chain at the protein level in guinea pigs. Also, multiple pseudogenes exist in the guinea pig V_H_ (413), V_κ_ (238), and V_λ_ (84) loci, which may contribute to the Ig diversity in guinea pigs, similar to other species [47], [88], [89].

In conclusion, we have reported the characterization and annotation of the guinea pig Ig loci genomic maps for the first time. This information, together with the characterization of the guinea pig Ig germline gene repertoire currently being undertaken, should lay the foundations for further studies into the differentiation and structure of mammalian Ig genes, including those found in guinea pigs.

## Supporting Information

Figure S1 **Multiple sequence alignments of guinea pig V_H_ genes.**
(DOC)

Figure S2 **Multiple sequence alignments of guinea pig V_κ_ genes.**
(DOC)

Figure S3 **Multiple sequence alignments of guinea pig V_λ_ genes.**
(DOC)

Figure S4 **Guinea pig immunoglobulin heavy chain constant region encoding gene amino acid sequences.** Four guinea pig encoding gene amino acid sequences of constant region (IgM, IgG, IgE and IgA) are cloned by 3′RACE.
(TIF)

Figure S5 **Analysis results of the guinea pig Ig μ, γ, ε and α genes in scaffold 54.** Four guinea pig constant region encoding genes of tansmembrane type for IgM, IgG, IgE and IgA were identified by bioinformatics analysis.
(TIF)

Figure S6 **Alignment of the IgG amino acid sequences of the guinea pig.** Alignment of IgG1, IgG2 and IgG squence of guinea pig. Dots indicate similar residues as in G1 and G, whereas dashes indicate gaps introduced for optimal alignment. The diference is represented by grey background between G and G1.
(TIF)

Figure S7 **Southern blotting analysis of guinea pig genomic DNA.** Southern blotting analysis of guinea pig heavy chain constant region IgG genes. The genomic DNA was digested with *Bam*H I (B), *Ec*oR I (E), *Hin*d III (H) and *Xba* I (X), and hybridized with probes for IgG-CH1 sequence.
(TIF)

Figure S8 **Guinea pig germline D_H_ segments.** The nonamers (9-mer) and heptamers (7-mer) are displayed. Heptamer components that are different from the consensus (5′: CACTGTG and 3′: CACAGTG) are shadowed. The deduced amino acids of all three reading frames of the coding region of D segments are shown, all the D_H_ segments were attached by 12 bp spacer.
(RAR)

Table S1 **Primers were designed for amplify guinea pig four classes immunoglobulin M, E, A, and G genes.**
(DOC)

Table S2 **GenBank accession numbers or references of the gene sequences from other species.**
(RAR)

Table S3 **The guinea pig immunoglobulin heavy chain DNA segments location in scaffolds 54 and 75.**
(RAR)

Table S4 **The guinea pig immunoglobulin κ light chain DNA segments location in scaffolds 37 and 24.**
(RAR)

Table S5 **The guinea pig immunoglobulin λ light chain DNA segments location in scaffold 4.**
(RAR)
