# Supplementary material for: Immunoglobulin Genomics in the Guinea Pig (Cavia porcellus)
Source: PLoS One. 2012 Jun 22;7(6):e39298. doi: 10.1371/journal.pone.0039298 (PMC3382241; doi:10.1371/journal.pone.0039298)
Supplement: Figure S1 — Multiple sequence alignments of guinea pig VH genes. (DOC) [file pone.0039298.s001.doc]

Figure S1

> VH3-48-54

;

;

EVLLVESGGGLVQSEGSLRLSCVTSGFTFSN--YYMHWIRQAPGKGLEWVGLIRNAAKSH

TTEYAASVKGRFTISRDDSKSTLYLQMTKLKPEDTAV-YYC

> VH3-35-54

;

;

EVQLVESGGGLVQPEGSLRLSCVTSGFTFSN--YYMHWICQAPGKGLEWVGLIRNAAKSH

TTEYAASVKGRFTLSRDDSKSTLYLQMTKLKPEDTAV-YYC

> VH3-23-54

;

;

EVQLVESGGGLVQPEGSLRLSCVTSGFTFSN--YYMYWIRQAPGKGLEWVGLIRNAAKSH

TTEYAASVKGRFTISRDDSKSTLYLQMTKLKPEDTAV-YYC

> VH3-42-54

;

;

EVQLVESGGGLVQPEGSLRLSCVTSGFTFSD--YYMHWIRQAPGKDLEWVGFIRNAAYSH

TTEYAASVKGRFTISRDNAKSTVYLQMTKLKPEDTAV-YYC

> VH3-2-54

;

;

EVQLVESGGGLVQPEGSLRLSCVTSGFTFSD--YYMHWIRQAPGKDLEWVGFIRNAAYSH

TTEYAASVKGRFTISRDDSKSTVYLQMTKLKPEDTAV-YYC

> VH3-9-54

;

;

EVQLVESGGGLVQPEGSLRLSCVTSGFTFSD--YYMYWIRQAPGKGLEWVALIRNADNSH

TTEYAASVKGRFTISRDDSKTTVFLQMTKLKPEDTAV-YYC

> VH3-167-75

;

;

EVQLVESGGGLVKPGESLRLSCVASGFTFSS--YWMNWIRQAPGKGLEWISAINSD--GS

STYYADSVKGRFTISRDNGKNTLYLQMSSLRPEDTAM-YYC

> VH3-166-75

;

;

EVQLMESGGGLVKPGESLRLSCVASGFTFSS--YWMNCIRQAPGKGLEWISAIYND--GS

STYYADSVKGRFTISRDNGKNTLYLQMSSLRPEDTAM-YYC

> VH3-170-75

;

;

EVQLVESGGDLVTPGESLRLSCVASGFTFSS--YWMIWIRQAPGKGLEWISGINSD--GS

RIYYADSVKGRFTISRDNGKNTLYLQMGSLRPEDTAM-YYW

> VH3-201-75

;

;

EVQLTQSGGGLVQPGESLRLSCVASGSTLSN--YWMDWVRKAPGKGLEWISAISNS--GS

-TYYADSVKGRFTISRDNGKNALYLQMSSLRPEDTAV-YYC

> VH3-184-75

;

;

EVQLTQSGGGLVQPGESLRLSCVASGSTLSN--YWMDWVRKAPGKGLEWISAISNS--GS

-TYYADSVKGRFTISRDNGKNALYLQMSNLRPEDTAV-YYC

> VH3-26-75

;

;

EVQLVESGGGLVKPGESLRLSCVASGFTLSN--YWMDWVRQAPGKGLEWISAIS-G--GS

-TYYADSVKGRFTISIDNGKNTLHLQMSSLTPEDTAL-YYC

> VH3-262-54

;

;

EVQLVESGGGLVQPGGSLRLSCLASGFTFSN--YGMSWIRQAPGKGLEWISAISDS--GA

STYYADSVKGRFTISRDNGKNLLYLQMSSLRPEDTAV-YYC

> VH3-114-75

;

;

EVQLVESGGGLVQPGGSLRLSCVASGFTFDD--YGMSWIRQAPGKGLEWISAISYS--GG

STYYADSVKGRFTISRDNGKNTLYLQMSSLRPEDTAT-YYC

> VH3-194-54

;

;

EPQLVESGGGLVQPGGSLKLSCVASGFTFSN--AGMHWIRQAPGKGLEWISVIWYD--GS

KMYYADSVKGRFTISRDNSKNTLYLQMSSLRPEDTAV-YYC

> VH3-189-54

;

;

AEQLVESRGDLVQPGSSLRLSCMASGFTFSN--YDMHWIRQSPGKELEWISVIWYD--GS

KTYYADSVRGQFTISRDNSKNTLYLQVSSLRPEDMAMHYYC

> VH3-197-54

;

;

EAQLVESGGGLVQPGGSLRLSCVAWGFTFSS--YAMNWIRQAPGKGLEWISVIWYE--GS

KMYYADSVKGGFTISRDNGKNTLYLQMSSLRPEDTAL-YYC

> VH3-176-75

;

;

EEQLVESGGGLVQPGGSLRLSCVASGFSFST--YDMGWIRQAPEKGLEWISLLSYG--GG

SPDYADSVKGRFTISRDNGKNMLYLQMNSLRAEDTAV-YYY

> VH3-185-54

;

;

EEQLVESGGGLVQPGGSLKLSCVASGFTFSS--YDMSWIRQAPGKGLEWISLISSG--G-

SIYYAGSVKGRFTISRDNGKNTLYLQMNSLRPEDTAV-YYC

> VH3-74-75

;

;

EEQLVESGGGLVQPGASLRLSCVASGFTFSS--YYMAWIRQAPGKGLEWVTWISNT--GG

STGYADSVKGRFTISRDNGKNTLYLQMNSLRAEDTAL-YYC

> VH3-37-75

;

;

EEQLVESGGGLVQPGASLRLSCVASGFTFSS--YYMAWIRQAPGKGLEWVTWIGNT--GG

SIGYADSVKGRFTISRDNGKNTLYLQMNSLRAEDTAV-YYC

> VH3-80-75

;

;

EEQLVESGGGLVQPGASLKLSCVASGFTFSS--YDMAWIHQAPGKGLEWVTWISYT--GG

STKYADSVKGRFTISRDNGKNTLYLQMNSLRSEDTAV-YYC

> VH3-150-75

;

;

EEQLVESGGGLVQPGGSLRLSCVASGFTFSS--YYMAWIRQAPGKGLEWITRIGNT--GG

STNYADSVKGRFTISRDNGKNTLYLQMNSLRAEDTAV-YYC

> VH3-89-75

;

;

EEQLVESGGGLVQPGGSLRLSCVASGFTFSS--YHMSWIRQAPGKGLEWITWIRSD--GG

STGYADSVKGRFTISRDNGKNTLYLQMNSLRAEDTAL-YYC

> VH3-30-75

;

;

EEQLVESGGGLVQPGGSLRLSCMASGFTFSS--YHMSWIRQAPGQGLEWITWIRND--GG

STNYADSVKGRFTISRDNGRNTLYLQMNSLRPEDTAV-YYC

> VH3-157-75

;

;

EEQLVESGGGLVQPGGSLRLSCVASGFTFSS--YYMNWIRQAPGKGLEWISMISND--GS

STKYADSVKGRFTISRDNGKNTLYLQMSSLRPEDTAV-YYC

> VH3-80-54

;

;

EEQLVESGGGLVQPGGSLKLSCVASGFTFSD--YWMSWIHQAPGKGLEWLTYINYD--GD

STYYADSVKGRFTISRDNSKNMLYLQMSSLKPEDTAV-YYC

> VH3-104-54

;

;

EEQLVESGGGLVQPGGSLKLSCVASGFTFSN--YWMSWIRQAPGKGLEWLTYINPD--GG

STYYADSVKGRFTISRDNSKNMLYLQMSSLKPEDTAV-YYC

> VH3-229-54

;

;

EEQLVESGGGLVQPGGSLKLSCMASGFTFST--YVMHWIRQAPGKGLEWISYISQT--GK

NINYADSIKGRFTISRDNEKNTLYLQMSSLKPEDTAL-YYC

> VH3-183-54

;

;

EEQLVESGGGLVQPGGSLKLSCMASGFTFSS--YAMSWIRQAPGKGLEWISAIGSS--GS

NTYYAGSVKGRFTISRDNGKNTLYLQMSSLRPEDTAV-YYC

> VH3-137-54

;

;

EEKLVESGGGLVPPGGSLKLSCVASGFTFSS--YGMSWVRQAPGKGLEWITSVGTK--G-

NTYYAESLKDRFTISRDNGKNTLYLQMSRLIPEDLAV-YYC

> VH3-35-75

;

;

EEQLVESGGGLVQPGGSLRLSCVASGFTFSS--YEMNWIRQSPGKGLEWISYISSG--SG

SIYYADSVKGRFTISRDNSKNTLYPQMSSLRAEDTAV-YYC

> VH3-102-75

;

;

EEQLVESGGGLVQPGGSLRLSCVASGFTFSS--YHMNWIRQSPGKGLEWISYINSG--SG

GIYYADSVKGRFTISRDNSKNTLYLQMSSLRAEDTAV-YYR

> VH3-255-54

;

;

EQQLVESGGGLVQPGASLRLSCVASGFTFSN--YGMDWFRQTPGKGLEWISYISSN--SG

TIYYADSVKGRFTISRDNSKSTLYLQMSSLRPEDTAM-YYC

> VH3-36-75

;

;

EEQVVEPGGGFMQPGGSLRLFCVASGFTISS--YHRSWTHQVPGKGLEWIADISS---SG

STYYADSVKGRFTISRDNGKNTLYLQMNSLRPEDTAM-YHC

> VH3-8-75

;

;

EEQVVEPGGGLLQPGGSLRLFCVASGFIISS--YHRSWTHQAPGKGLEWIADISS---SG

GTYYADSAKGRFTISRDNSKNTLYLQMSSLRPEDTAM-HHC

> VH3-190-75

;

;

KVRLVESEGGLVQPGGSLRLSCVASGFTFSS--YGMDWINQAPRKVLEWISAISSN--SK

NTYYADSVKGRFTISRDNGKNTLYLQMSSLRLDDTAM-YYC

> VH3-186-54

;

;

EEQLVESGGGLVQPGSSLRLSCVASGFTFSS--YTMYWIRQAPGKGLEWISYISSS--SS

NIKYADSVKGRFTISRDNGKNTVYLQMSSLRPDDTAM-YYC

> VH3-286-54

;

;

EEQLVESGGGLVQPGGSLRLSCVASGFTFSN--YWMNWIRQAPGKGLEWISEINGD--SS

TINYIDSVKGRFTISRDNSKNTLYLQMSSLRSEDTAV-YYC

> VH3-271-54

;

;

EEQLVESGGGLVQPGGSLRLSCVASGFTFSN--YWMNWIRQAPEKGLEWISEINGD--SS

TINYIDSVKGRFTISRDNSKNTLYLQMSSLRSEDTAV-YYC

> VH3-230-54

;

;

EVQLMESGGGLVQPGGSLRLSCVASGFTFSD--YWMSWIRQAPGKGLEWISEIKGD--SS

TINYIDSVKGRFTISRDNSKNTLYLQMGSLRTEDTAV-YYC

> VH3-204-54

;

;

EPQLVESGGGLVQPGGSLRLSCVASGFTFSN--SWMDWIRQAPGKGLEWISEIKGD--SS

TINYIDSVKGRFTISRDNGKNTLYLQMSSLRPEDTAV-YYC

> VH3-265-54

;

;

DVQLVESGGGLVQPGGSLRLSCVASGFTFSN--YYMYWVRQAPGKGLEWLAAISGD--SS

NIKYADSVKGRFTISRDNSKNTLYLQMSSLRTEDTAV-YYC

> VH3-194-75

;

;

EVQLVESGGGLVPPGGSLRLSCVASGFTFSN--YYMHWIRQAPGKGLEWLAYISGD--SS

NIKYADSVKGRFTISRDNGKNTLYLQMSSLRTEDTAV-YYC

> VH3-212-75

;

;

EVQLVEAGGGLVQPGGSLRLSCVASGFTFSN--YYMHWIRQAPGKGPEWLTYISGD--SS

YIKYADSVKGRFTTSRDNGKNTLYLQMSSLRTEDTAV-YYC

> VH3-282-54

;

;

EVQLVESGGGLVQPGGSLRLSCLASGFTFSN--SWMSWVRQVPGKALEWLTNINGD--SS

NIKYADSVKGRFTISRDNGKNTLYLQMSGLRTEDTAL-YYC

> VH3-222-54

;

;

ELQLVEYGGSLVPPGGSLTLSCVTLEFPFSS--YAMGWIRQPPGQGLEWLSLIYHD--SS

KINYANSVKGRFTISRDNGRNTLYLQMNSLRTEDTAL-YYC

> VH3-63-75

;

;

EEQLVESGGGLVQPGGSLRLSCVASGFTFSD--YYMSWIRQSPGKGLEWIAGINDD--SS

DTQYANSVKGRFTISRDNGKNTLYLQMSGLRPEDTAM-YYC

> VH3-3-75

;

;

EEQLVESGGGLVQPGGSLRLSCVASGFTFSD--YYMRWIRQSPGKVLEWIAGINDD--SS

DTQYANSVKGRFTISRDNGKNTLYLQMSSLRPEDTAV-YYC

> VH3-139-75

;

;

EEQLVESGGGLVQPGGSLRLSCVASGFTFSN--WYMSWIRQAPGKGLEWIAAISDD--SS

DIEYANSVKGRFTISRDN-NNTLYLQMSGLRPEDTAV-YYC

> VH3-51-75

;

;

EEQLVESGGGLVQPGGSLKLSCVASGFTFSD--YYMSWIRQAPGKGLEWIAGISSS--SS

YIEYADSVKGRFTISRDNGKNTLYLQMSSLRTEDTTM-YYC

> VH3-24-75

;

;

EEQLVESGGGFILPGGSLRLSCVASGFTFSN--YGMSWIRQAPGKGLKWFSGISSS--SS

YIDYADTLKGRFTISRDNGKNTLYLQMSSLRAENTVI-YYS

> VH3-98-75

;

;

-VQLVESGGGLVQPGGSLRLSCMASGFTFSN--YWMSWIRQAPGKGLEWIAGIDSS--SS

YIQYAYSVKGRFTISRDNGKNTLYLQMSGLRAQDSAA-YYC

> VH3-20-75

;

;

DEQLVESGGGLVQPGGSLRLSCVASGFTFSN--YYMHWIRQAPGKGLEWISRISSS--SS

YIDYADSVKGRFTISRDNSKNTLYLQMSSLRPEDTAV-YYC

> VH1-75-54

;

;

QVQLQESGSGLVKPSQTLSLTCSVSGFSITTGSYEWSWIRQTPGKSLEWMGYISSN---G

GTSYNPSFKNRISISRDTGKNQFSLQLNSVNTEDTAT-YYC

> VH1-66-54

;

;

QVQLQESGSGLVKPSQTLSLTCSVSGFSITTGSYEWSWIRQTPGKSLEWMGYISSN---G

GTSYNPSFKNRISISRDTGKNQFSLQLNSVNTEDTAT-YYC

>VH1-84-54

;

;

QVQLQESGTGLVKLSQTLSLTCIVSGFSITTSSYEWHWIRQPPGKSLEWMGAIGSS---G

GTGYNPSLKSRISISRDTGKNQFSLQLNSVTAEDTAT-YYC

> VH1-68-54

;

;

QVQLQESGSGLVKPSQTVSLTCSVSGFSITTTNYWWSWIRQPPGKSMEWMGYIDYI---G

GTSYNPSLQSRLSISRDTGKNQFSLQLNSVTTEDTAT-YYC

> VH1-95-54

;

;

QVQLQESGSGLVKPSQTLSLTCSVSGFSITTTNYWWSWIRQPPAKSLEWMGHISYS---G

STYYNPSFQSRISISRDTGKNQFSLQLNSLTTEDTAT-YYC

> VH1-59-54

;

;

QVQLQESDSGLVKPTQTLSLTCSVSGISITTSYYCWNWMRQPPGKSLEWMGYICYS---G

STGYNPSLQSRISVSRDTGKNQFSLKLNSVTTEDTAI-YYC

> VH1-91-54

;

;

QMQLQESGPGLVKPSQTLFLTCSVSGFSITTSGYGWSWIRQPRGKTLEVMGGIAYN---G

GTGYNPSIKSRISISRDTGKNQFSLQLNSVTEEDTAT-YYC

> VH1-14-54

;

;

QLQLQESGPDLMKPSQTLTLTCLVSGYSITS-DSTWKWIRQSPGKSLQWMGHIWYD---G

DTKYNPTLQSRISISRDTGKNQFSLKLNSVTTEDTAT-YYC

> VH1-4-54

;

;

QLQLKESGPDLVKPSQTLFLTCSGSGYSITS-DNAWIWIPQPPGKSLQWMGHVWYD---G

DTKYNPTLQSRISISRDTGKNQFSLQLNSVTTEDTAT-YYC

> VH1-140-54

;

;

QVQLQESGPGLVKPSQTLSLTCTVSGYSITS-GYDWAWIRQPPGKSLEWMGYISSG---G

STRYNPSLQSRMSISRDTGKNQFSLQLKSVTAEDTAT-YYC

> VH1-120-54

;

;

QVQLQESGPDLVKPSQTLSLTCTVSGYSITS-DYYWAWIRQPPGKGLEWMGYIRYS---G

STEYNLSLQSRMSISRDTGKNQFSLQLKSVTAEDTAT-YYC

> VH1-115-54

;

;

QVQLQESGPDLVKPYQALSLTCSVSGYSITS-GYSWNWIRQPPGKSLEWMGYISSS---G

GTGYNPSLQSRISISRDTGKNQFSLQLKSITAEDTAT-YYC

> VH1-77-54

;

;

QVQLQESEPSLVKPSQTLSLTCSVSGFSITTSYYYWNWVRQPPGKSLEWMGYIRYD---G

STNYYPSFKRRVSISRDKGKNQFSLRLTSVTAEDTAT-YYC

> VH1-101-54

;

;

QVQLQESEPSLVKPSQTLSLTCSVSGFSITTSYYYWNWVRQPPGKSLEWMGYIRYD---G

STNYYPSFKRRISISRDKGKNQFSLRLTSVTAEDTAT-YYC

> VH1-151-54

;

;

QVQLQESGPGLVKPSQTLSLTCTVTGFSITTSSYYWSWIRQPPGKSPEWMGVIDYD---G

DTAYSPSLKSRISISRDTGKNQFSLQLRSVTPEDTAT-YYC

> VH1-171-54

;

;

QVQLQESGSGLVGPSQTLSLTCSVSGFSITTNGYAWGWIRQPPGKSLEWMGHIWYD---A

DTYYNPSIKNRISISRDTGKNQFSLQLSSITTEDTAT-YYC

> VH1-58-54

;

;

QVQLQESGPGLVKPSQSLSITCTVSGFSITTTNYWWHWICQPPGKSLEWLGEISYD---G

NTKYNPSVKSRLSIARDTGKNQFSLQLTSVTTEDTAT-YYC

> VH1-1-54

;

;

QVQLQESRPGLVKPSQNLSLTCSVSGLSITNSHYWWDWIRQPPGKSLEWMGVISYD---D

STGYNPSVKSRISISRDTGKNQFFLQLTSVTTEDTAI-YYC

> VH1-56-54

;

;

QVQLQELGPGMVKPSQTLSLTCLDSGLSITTSGSYWSWICHPLGKSLEYMGYLHSS---G

SPNYNPSVKSQFSIFRDIDRKQSSLQLNSVTGEDTAT-YYC

> VH1-156-54

;

;

QVQLQESGSGLVKPSMTLSLTCSVSRFSITTGNYWWHWICGPPRKDPRVDGHITNS---G

STRYNPSLHGHLSIFRDTGKTHFSLQLNSVTTEDSVI-YYH

> VH1-215-54

;

;

QVQLQESGLGLVMPSQTLSLICTVSGSSITS-GYYWSWIRQIPGKSLEWMGYIYPS---S

STSYSPSLQSRISISIDTGKNQFSLQLNSVTAEDTAT-YYC

> VH1-209-54

;

;

QVQLQESGLGLVMPSQTLSLTCTVSGSSITS-GYYWSWIHQIPGKSLEWMGYIYPS---R

STSYSPSLQSRISISIDTGKNQFSLQLNSVTAEDTAI-YYC

> VH2-94-75

;

;

QVQLQESGPGLVKPSETLSLTCTVSGFSLTS--YSVHWVRQAPGKGLEWIGAIWSD---G

STYYNSALKSRVGISRDTSKSQVSLTLSSLSPEDTAV-YYC

> VH2-54-75

;

;

QVQLQESGPGLVKPSETLSLSCTVSGFSLTS--YSVYWVRQAPGKGLEWIGAIWSG---G

STDYNSALKSRVRISRDTSKSQVSLTLSSLSPEDTAV-YYC

> VH2-17-75

;

;

QVQLQESGPGLVKPSETLSLTCTVSGFSLTS--YSVSWVRQAPGKGLEWIGRIWSD---G

STDYNSALKSRVGISRDTSKSQVSLTLSSLSPEDTAM-YYC

> VH2-7-75

;

;

QVQLQESGPGLVKPSETLSLTCMVSGFSLTS--YSVSWVRQAPGKGLEWIGRMWSG---G

STDYNSAFKSRVGISRDTSKSQVSLTLSSLSPEDTAM-YYC

> VH2-240-75

;

;

QVQLQESGPGLVKPSETLSLTCTVSGFSLTS--AGVNWVRQAPGKGLEWIGGIWSG---G

STDYNSALKSRVGISRDTSKSQVSLTLRSVSPEDTAV-YYC

> VH2-67-75

;

;

QVKLQESGPGLVKPSETLSLTCTVSGFSLTS--YGVHWVPQAPGKWLEWIGVIWDG---G

STDYNSAFKYRVGISRDTSKKQASLTLSSLSPEDKAM-YYC

> VH2-27-75

;

;

QVQLQESGPGLVKPSETLSLTCTVSGFSLTS--YGVHWVRQAPGKGLEWIGVIWGG---G

STDYNSAFKSRVGISRDTSKSQASLTLSSLSPEDTAV-YYC

> VH2-43-75

;

;

QVQLQESVPGLVKPSETLSLTCTVSGFSLTN--YGVHWIDQAPGKGLEWIGAIWSG---G

STDYNSAFKSRVGISRDTSKSQASLTLSSLSPQDTAV-YYC

> VH2-35-75

;

;

QVQLQESGPGLVKPSETLSLTCKVSGFSLTG--YSVSWIRQAPGKGLEWIGAIWSF---G

STDYNSAFKSRVGISRDTSKSQVSLTLSSLSPEDTAV-YYC

> VH2-160-75

;

;

QVQLQESAPGLLKPSETLSLTCMISGFSLTC--CGVSWVHQSPGKGLEWIGGIWVC---G

STDYNSALQSRVGIRRGTLKSQVSLILSSLSPEDMAV-HYC

> VH2-86-75

;

;

QVQLQESGPGLVKPSETLSLTCTVSGFSVSS--YGVHWVRQPPGKGLEWIGVIWSY---G

STNYNSALQSRVGISRDTSKSQVSLTLSSLSLEDTAV-YYC

Figure S1

> VH2-121-75

;

;

QGQLQESGPSLVKPSETLSLTCTVSGFSLTS--NGFSWVRQAPGKGLEWIGVIANN---G

GTIYNSALQSRVGISRDTYKSQVSLTLRNLIPEDTAM-YYC

> VH2-109-75

;

;

QGQLQESGPGLVKPSETLSLTCTVSGLSLTS--NAFNWVRQAPGKGLEWIGVIWSG---G

STDYNPALKSRVGISRDTSKSQVSLTLSSLSPEDTAM-YYC

>VH2-70-75

;

;

QVQKQESGPGLVKPSETLLLTCTGSGFSLSS--YHAHWVRQAPGKGLEWIGAIWSG---G

STDYNSAFKSRVGISRDTSKSQASLTLSSLSPEDTAV-YYC

> VH2-28-75

;

;

QVQLQESGPGLVKPSETVLLTCTASGFSLSS--NHAHWVRQATGKGLEWIGAIWSG---G

STDYNSAFKSRVGISRDTSKNHVSLTLSSLSPEDTAM-YYC

> VH2-145-75

;

;

QMQLQESGPHLVKPSETLLLTCTVSGFHLTS--YDVQWVRQSPGKGMEWIGAIWSG---G

STDYISALKSRVGISRDTSKSLVSLTMRSLSPKDTAV-YYC

> VH2-216-75

;

;

QMQLQESGPNVVKPSETRSLTCTVPGFSVIS--YHVHWVWQATGKGLEWIGAIWES---G

GTSYTSAIESRVTISKETSKNQVSLTLSILSPEDTAM-YYS
