# Supplementary material for: Immunoglobulin Genomics in the Guinea Pig (Cavia porcellus)
Source: PLoS One. 2012 Jun 22;7(6):e39298. doi: 10.1371/journal.pone.0039298 (PMC3382241; doi:10.1371/journal.pone.0039298)
Supplement: Figure S2 — Multiple sequence alignments of guinea pig Vκ genes. (DOC) [file pone.0039298.s002.doc]

Figure S2

> VK1-281

;

;

GACATCCAATTGACACAGCCTGCATCTGCA---TCTGCATCTGTGGGAGACACAGTCAAG

ATCAGTTGCCGGGCCAGTTTAAACTGGTATCAGCAGAAACCAGGGCAAGCTCCTAAACTC

CTGATCTATAATTTGCAGTCTGGGGTCCCATCGAGGTTCAGTGGCAGTGGATCTGGGACA

GATTTCACTCTCACCATCAGCAGCCTGAAGCCTGAAGACTTTGCAACTTATTACTGT

> VK1-246

;

;

GACATCCAGTTGACCCAGTCTCCATCCATCCTGTATGCATCTCCAGGAGACAAAATCACC

ATTACTTGTCGGGCCAGTTTAGGCTGGTATCAGCAGAAACCAGGGAACGCCCCTGAGCTC

CTGATCTATAATTTGTACGCTGGGGTCCCAGCAAGGTTCAGTGGCAGTGGATTTGGGACA

GATTTTACTCTCACCATAAGCAGCTTGGAATGTGAGGATTCTGCAATTTATTACTGT

> VK1-233

;

;

GACATCAAATTGACTCAGCCAGCATTGATA---TCTGCATCTGTGGGAGACACAGTCATA

ATCAGTTGTAGGCCCAGTTTAAGCTGTTATCACCAGAAACCAGGGCAAGCTCCTAAACTC

CTGATCTTTAGTTTGCAGTCTGGAGTCCCATCAAGATTCAGTGGCAGTGGGTCTGGGACC

GATTTCACTCTCACCATTCGTAGCCTGCAGCCTGAAGATCTTGCAACTTATTACTGT

> VK1-133

;

;

GACATCCAGATGATCCAGTCACCAGCTTTCCTGTCTGCATCTCTAGGAGACACAGTCACC

ATTAATTGCTGGGCCAGTTTAGCCTGGTATCAGTGGAAACCAGGGCAAGCTCCTAAACTC

CTGATCTACAATTTGCAATCTGGGGTCCCATCAAGGTTCAGCGGCAGTGGATTTGGGACA

GATTATTCTCTTACCATCAGAAGACTGGAAACTGAAGATGTGGCAACTTATTACTGT

> VK1-130

;

;

GACATCCAGATGACTCAGTCTCCCTCCTCCATATCTGCATCCATAGGAGAGAGAGTCACC

ATCAGTTGCAGGGCAAGTTTAGCTTGGTATCAACAGAAACCAGGGAAAGCTCCTAAACTA

CTGATCTACAAGTTGGAATCCGGGGTCCCCTCTCGGTTCAGTGGCAGTGGAGCAGGGACA

GATTTCACTCTCACCATCAGCAGTCTGGAACCTGAGGATGTCGCAACTTACTACTGT

> VK1-128

;

;

GACATCCAGATGACTCAGTCTCCCTCCTCCATACCTGCATCCATAGGAGAGAGAGTGACC

ATCAGATGCAGGGCAAGTTTAGCTTGGTATCAACAGAAACCAGGGAAAGCTCCTAAACTA

CTGATCTACAAGTTTGAATCCGGGGTCCCCTCTCGGTTCAGTGGCAGTGGAGAAGGGACA

GATTTCACTCTCACCATCAGCAGTCTGGAACCTGAGGATGTTGCAACTTACTACTGT

> VK4-95

;

;

GACATTGTGATGACCCAGTCTCCAGCCACCATGGCTGTGTCGCCTGGAGAGAGAGTCACC

ATCCACTGCAAGTCCAGCTTAAGCTGGTACCAGCAGAAACCAGGCCAATCTCCCAAACTG

CTCATCTATACCCGAGCATCTGGGGTCCCAGACCGGTTCAGTGGCAGTGGGTCTGGCACC

GATTTCACTCTCACCATCAGCAGCCTGCAGGCTGAAGATGTGGCAGATTATTTCTGC

> VK4-90

;

;

GACATTGTGATGACCCAGTCTCCAGCCTCCCTGGCAGTGACTCCTGGAGAAAGCGCCACC

ATCCACTGCAAGTCCAGCTTAGCCTGGTACCAGCAGAAACCAGGGAAATCTCCCAAACTG

CTCATCTTTACCCGGGCATATGGCATCCCAGACCGGTTCACTGGCAGTGGGTCTGGCAGG

GATTTCACCTTGACCATCAGCAGGGTGCAGGCTGAAGATGTGGCAGATTATTACTGT

> VK2-85

;

;

GACATTAGGATGACCCAGACCCCACTCTCTCTGTCTGTCTCCCCTAGAGAGCCAGCCTCC

ATCTCCTGCAGCTCCAGTGTCTATTGGCTTGTGCACAAGCCAGGCAGGGACAAACAGGGC

TTCATTTATACCCTGAATTCTGGCATCTCAGATAGGTTCAGTGGCAGTGGGTCAGGGACA

GATTTCACACTCAAAATCAGCAGGGTGGAGGCTGAGGATGCTGGAGTTTATTACTGT

>VK5-57

;

;

CAAATTGTGCTCACCCAGACTCCAGCATCCCTGGCTGCTTTTCCAGGGGAGAAGGTCACC

ATCACCTGCATAGTCAGGTTGCACTGGTACCAACAAAAGGCAGGAGCCTCTCCCAAGCTC

CTAATTTATGAGCTGGCCTCGGGAGTCCCAGCTCGCTTCAGTGGGAGTGGGTCAGGGACT

TCTTTCTCTCTCACAATCAGCAACGTGGAGGCTGAAGATGTTGCAACCTATTACTGT

> VK2-50

;

;

GATGTTGTGGTGACCCAGACCCCACTCTCTCTGTCCATCGCCCCTGGAGAGCCAGCCTCC

ATCTCCTGCAAGGCCAGTTTGAACTGGGTTGTGCATAAGTCAGGCCAGGCTCCACGACGC

ATGATTTATAATCGAGACTCTTGGGTCTCTGACAGGTTCAGTGGCACTGGGTCAGGCACA

GATTTCTCTCTCAAAATTAGCAGGGTGGAAGCTGAGGATGCTGGAGTTTATTACTGT

> VK4-32

;

;

GACATTGTGATGACCCAGTCTCCAGCCTCTCTGGCTGTGTCTCCTGGAGAGAGAGTCACC

ATCCACTGCAAGTCCAGCTTAAGCTGGTACCAGCAGAAACCAGGCCAATCCCTGAAACTG

CTCATCTACACCCAGGCATCTGGGGTCCCAGACCGGTTCAGTGGCAGTGGGTCTGGCACC

GATTTCACTCTCACCATCAGCAGCCTGCAGGCTGAAGATGTGGCAGATTATTTCTGC

> VK4-29

;

;

GACATTGTGATGACCCAGTCTCCAGCCTCCCTGATTGTGTCTCCTGGAGAGAGCGCCACC

ATTAGGTGTCAGTCCAGTTTAAGCTGGTACCAGCAGAAACCAGGACAATCCCCCAAACTG

CTGATCTACACCCGAGCATCTGGAATCCCAGAGCGGTTCAGTGGCAGTGGGTCTGGCACG

GATTTCACTCTCACCATCAGCGGCGCACAGGCTGAAGATGTGGCGAGTTATTACTGT

> VK4-28

;

;

GACATTGTGATGACTCAGTCTCCAGCCTCCCTGTCAGTGACCCCTGGAGAGAGCACCACC

ATCCGCTGCAAGTCCAGCTTAGCCTGGTACCAGCAGAAACCAGGGCAATCTCCCAAACTG

CTCATCTACACCCGAGACACTGGGGTACCAGACCGGTTCAGTGGCAGTGGGTCTGGTACC

GATTTCACTCTCACCATCAGCAGCGTGCAGGCTGAAGATGTGGCTGATTATTACTGT

> VK3-280

;

;

CAAACCCAGCTGACTCAGACTCCAGAATCCCTGGCTGTGTCTCTTGGAGAAACAGTCACT

CTCAGCTGCAGGGCCAGTCTAGAATGGTACCAGCAGAAACCTGGGCAGGCTTCCAGACTC

CTCATCTATAGCAGGGTCACTGGTGTCCCAGCCCGGTTCAGTGGCAGCGGATCAGGGACA

GACTTCACTCTCACCATCAGCAGCCTGCAGCCCGAGGACATTGCAGTTTATCACTGT

> VK1-279

;

;

GACATCCAGATGACTCAGACTCCATCTTCCTTGCCTGCATCTGTGGGAGACACAGTCACC

ATCAAGTGTAAGGCCAGTTTAAACTGGTTCCAACAGAAACCCGGGAAAGCTCCTCAACTG

CTGATCTATAGTTTGGGATCTGGGGTTCCTTCAAGGTTCAGTGGCAGTGGATTTGGGACA

GATTTCACTCTTGCCATTAGTAGCCTACAGCCTGAAGATGTTGGTACCTACTACTGT

> VK1-278

;

;

GACATCCAATTGACACAGCCTGCATCTGCA---TCTGCATCTGTTGGAGACACAGTCAAG

ATCAGTTGCCGGGCCAGTTTAAACTGGTATCAGCAGAAACCAGGGCAAGCTCCTAAACTC

CTGATCTACAGCTTGGCATCTGGGGTCCCATCGAGGTTCAGTGGCAGTGGTTCTGGGACA

GATTTCACTCTCACCATCAGCAGCCTGCAGCCGCAAGACTTTGCAGTTTATTACTGT

> VK1-274

;

;

GACATCCAGTTGACACAGCCTGCATCTGCA---TCTGCATCTGTGGGAGACACAGTCAAG

ATCAGTTGCCGGGCCAGTTTAAGCTGGTATCAGCAGAAACCAGGGAAAGCTCCTCAACTG

CTGATCTACAGCTTGGCATCTGGGGTCCCATCGAGGTTCAGTGGCAGTGGTTCTGGGACA

GATTTCACTCTCACCATCAGCAGCCTGCAGCCGCAAGACTTTGCAACTTACTACTGT

> VK1-271

;

;

GACATCCAGATGACTCAGACTCCGTCTTCCTTGCCTGCATCTGTGGGAGACCCAGTCACC

ATAAAGTGTAAGGCCAGTTTAAACTGGTACCAACAGAAACCAGGGAAAGCTCCTCAACTG

CTGATCCATAATTTGGGATTTGGGGTTCCTTCGAGGTTCAGTGGCAGTGGATTTGGGACA

GATTTCACTCTTGCCATCAGTAGCCTACAGCCTGAAGATGTTGCTACCTATTACTGT

> VK1-270

;

;

GACATCCAGTTGACACAGCCTGCATCTGCA---TCTGCATCTGTGGGAGACACAGTCAAG

ATCAGTTGCCGGGCCAGTTTAAACTGGTATCAGCAGAAACCAGGGCAAGTTCCTAAACTC

CTGATCTATAGCTTGGCATCTGGGGTCCCATCGAGGTTCAGTGGCAGTGGTTATGGGACA

GATTTCACTCTCACCATCAGCAGCCTGCAGCCTGAAGACTTTGCAACTTACTACTGT

> VK1-266

;

;

GATATCCAGATGACTCAGGCTCCCTGGTCCCTATCTGCATCTGTAGGAGACAAGGTCACC

ACCACTTGCAAGGCCAGTTTCAACTGGTACCAACAGAAAGCAGGGAAAGCTCCAAAACTG

CTCATATATAATTTGAAATCTAGGATCCCGTCGCGGTTCAAAGGCAGTAGATCTGGAACA

TATTTCTTTCTCACCATCAGCAGCCTGCAGCCTGAAGATGCTGCGACTTATTACTGT

> VK3-263

;

;

CAAAAATTACTGACTAAGACTCCAGCTTCCCTGGCTGTGTCTCCCAGACAAACTGTCACT

TTCAGCTGCAGGGCCAGTTTAGAATGGTACCAGCAGAAACGTGAGCAGGTTTCCAGGCTC

CTCATCTATAGCAGGACCACTGGCATTCCAGGCCGGTTCAGTGGAAGTGGGTCAGGGACA

GACTTCACTCTCACCATCAGCAGCCTGCAGCCTGAGAATGTTGCAGTTTATCACTTT

> VK1-261

;

;

GACATCCAACTGACACAACCTGCATCCTTA---TCTGCATCTGTGGGAGACACAGTCAAA

ATCACTTGCCAGGCCAGTTTAAACTGGTATCAGCAGAAAGCAGGACAACCTCCTAAACTC

CTGATCTACGATTTGCAGTCTGGGATCCCATCAAGGTTCAGTGGCAGTGGATCTGGGACA

GATTTCACTCTCACCATCAGCAACCTGCAGCCTGAAGACTCTGCAACTTATTACTGT

> VK1-254

;

;

GACATTCAGATGACCCAGTCTCCATCTTCCATATCAGCATCCTTAGGAGAGAAGGTTACC

ATCAGTTGTCGGGCAAGTTTATCCTGGTATCAGCAGAAACCTGGGAAAGCTCCTAGACTT

CTGATCTATAATTTGGACACTAGGATCCCATCGAGGTTCAGTGGTAGTGGATCTGGGACA

GATTTCACTATCACCATCAACAGCCTCCAGTCTGAAGATT------CTTATTACTGT

> VK1-253

;

;

GACATCCAGTTGATGCAGCCTCCATCTGCA---TCTGCATCCGTGGGAGACACAGTCAAG

ATCAGTTGCCGGGCCAGTTTAGACTGGTATCAGCAGAAAGGAGGACAAGCTCCTACACTT

CTGATCTATAGATTGCAGTCTGGGGTCCCATCGAGGTTCAGTGGCAGTGGATCTGGGACA

GATTTCACTCTCACAATCAGCAGCCTGAAGCCTGAAGACTTTGCAACTTACTACTGT

> VK1-242

;

;

GACATCCAGTTGACACAGCCTCCATCTGCA---TCTGCTTCTGTGGGAGACACAGTCAAG

ATCAGTTGCCGGGCCAGTTTAAACTGGTATCAGCAGAAACCAGGGCAAGCTCCTAAACCC

CTGATCTATAATTTGCAGTCTGGGGTCCCATCAAGGTTCAGTGGCAGTGGATCTGGGACA

GATTTCACTCTCAGCATTAGCGGCCTGCAGCCTGAGGACTTTGCAACATACTACTGT

> VK1-239

;

;

GACATCCAGTTGACACAGCCTCCATCTGCA---TCTGCTTCTGTGGGAGACACAGTCAAG

ATCAGTTGCCGGGCCAGTTTAAACTGGTATCAGCAGAAACCAGGGCAAGCTACTAAACCC

CTGATCTACAGCTTGGAATCTGGGGTCCCTTCGAGGTTCAGTGGCAGTGGATCTGGGACA

GATTTCACTCTCAGCATTAGCGGCCTGCAGCCTGAGGACTTTGCAACATACTACTGT

> VK1-234

;

;

GACATACAGATGACCCAGTGTCCATCCGCCCTGTCTGTATCTCCTGGGGACACAGTTACC

ATCAATTTCAGAGCCAGTTTAAATTGGTATCAGCAAAAACCAGGGAAAGCTCCTACGCTC

CTGGTCTATAATTTGCAGTCCAGGGTCCCAATCAGGTTCATTGGCAGTAGATTTGGGACA

GATTTCACTCTCACCACCAGCAGCCTGCAGCCTGAAGATGTTGCATCTTATTTCTGT

> VK1-230

;

;

AATATCCAGATGACTCAGGCTCCTTCGTCCTTATCTGCTTCTGTAGGAGACAAGGTCACC

ATCACTTGCAAGGCCAGTTTAAACTGGTACCAACAGAAACCAGGGAAAGCTCCTCAACTG

CTGATCTCAAATTCGGAATCTGGGATCCCAGTGAGGTTCAGTGGCAGTGGATCTGGGACA

GATTTTACTCTCAACATCAGCAGCCTGCAGCCTGAAGATGTTGTGATTTATAATACA

> VK1-226

;

;

GACATCCAGATGACTCAGACTCCATCCTCCTTGCCTGCATCTGTGGGAGACACAGTCACC

ATCAAGTGTAAGGCCAGTTTAAATTGGTACCAACAGAAACCAGGGAAAGCTCCTCAACTG

CTGATCTATAACTTGGGATCTGGGGTCCCATCGAGGTTCAGAGGTAGTGGATCTGGGACA

GATTACACTCTCACCATCAGTAGCCTGCAGCCTGAAGATGTTGCTATTTATTACTGT

> VK1-255

;

;

GACATCCAGTTGACACAGCCTGCATCTGCA---TCTGGGTCTGTGGGAGACACAGTCAAG

ATCAGTTGCCGGGCCAGTTTAAACTGGTATCAGCAGAAACCAGGGCAGGCTCCTAAACTC

CTGATTTACAATTTGCAATCTGGGATTACATCAAGATTCAGTGGCAGTGGATCTGGGGCA

GATTATACTCTCACCATCACTGGCCTGCAGCCTGAGGACTTTGCAACTTATTTCTGT

> VK1-219

;

;

GACTTCCAGATGACCCAGTCACCATCTTCCTTGTCTGTATCTCCCGGAGACAGTGTCACC

ATCACTTGCAGAGCCAGTATAAATTGCTATCAGCAGAAACCAGGGAAAGCTCCTAAACTT

CTGATATATAATTTGCAATCTGGTGTCCCAACCAGGTTCAGTGGCAGTGGATCTGGTACA

GATTACACTCTTACCATCAGTAGCTTGCAGCCTGAAGATGTTGCATCTTATTACTGT

> VK1-214

;

;

GACATCCAGTTGACACAGCCTCCTTCTGCA---TCTGCATCTGTGGGAGACACAGTCAAG

ATCAGTTGCCAGGCCAGTTTAAACTGGTATCAGCAGAAACCAGGGCAAGCTCCTAAATGC

CTGATCTATAATTTGGAGTCTGGAGTCTCAACTAGGTTCAGTGGCAGTGGATCTGGGACA

GATTTCACTCTCACCATTAGCAGCCTGCGGCCGGAAGATTTTGCAGTTTATTACTGT

> VK1-211

;

;

GACATCCAGTTAACACAGCCTCCATCTACA---GCTGCATCTATGGGAGAAACAGTCACC

ATCAGTTGCAGGGCCAGTTTAAATTGGTATCAGCAGAAACCAGGGCAGGCTCCCAAACTT

CTGATCTACAGGTTGAAATCTGGGGTCCCATCAAGGTTCAGTGGCAGTGGATATGGGACA

GATTTTACTCTCACCATCAGTAGCCTGCAGCCTGAAGACTTTGCAATTTACTACTGT

> VK1-203

;

;

GACATCCAACTGACACAACCTGCATCCTTA---TCTGCATCTGTGGGGGACACAGTCAAG

ATCAGTTGCCAGGCCAGTTTAAACTGGTATCAGCAGAAACCAGGACAACCTCCTAAACTC

CTGGTCTACATTTTGCAATCTGGGCTCCCATCAAGGTTCAGTGGCAGTGGATCTGGGAAA

GATTTCACTCTCACCATCAGCAACTTGCAGCCTGAAGACTCTGCTACTTATTACTGT

> VK1-201

;

;

GACATCCAGTTGACGCAGCCTCCATCCAAA---TCTGCATCTGTGGGAGACACAGTCACC

ATCAGTTGTCAGGCCAGTTTAAACTGGTATCAGCAGAAACCAGGGCAAGCTCCTAAACTC

CTGATCTATAATTTGCAGTCTGGAATCCCATCGAGGTTCAGGGGCAGTGGATCTGGGACA

GATTTCACTCTCAGCATCAACAGCCTGCAACCTGAAGACTATGCAACTTATTACTGT

> VK1-190

;

;

GACATCCAGATGACCCAGTCACCATCTTCCCTCTCTGTATCTCCTGGAGAGAGAGTCACC

ATCACTTGCAGAGCCAGTTTAAATTGGTATCAGCAGAAACCAGGGAAAGCTCCTAAACTC

CTGATCTATAATTTGGTACCCGGGGTCCCATCCAGGTTCAGTGGCAGTGGATCTGGAACA

GATTACAGTCTCACAATCAGTTCTGTGGAAGCTGAAGATATTGCAACTTATTACTGT

> VK1-184

;

;

GACATTCAGATGAGCCAGTCTCCATCCTCCCTCTCAGTATCTCTTGGAGACAGAGTCACC

ATCACTTGCAAAGCTAGTTTGGCCTGGTATCATCAGAAACCAGGGAAAGCTCCTAGGATC

CTCATCTATGATTTGTTTGCCCTAGTCCCATCCAGGTTCAGTGGCAGTGGATCTAGTACA

GATTACACTTTGGCTATCAGCACAGTGGAGGCTGAAGATGTTGGAACTTACTACTGT

> VK1-179

;

;

GACATCCGGATGACTCAGACTCCATCCTCCTTGCCTGCTTCTGTGGGAGACACAGTCACC

ATCAAGTGTAAGGCCAGTTTAAGTTGGTACCAACAGAAACCAGGGAAAACTCCTCAACTG

CTGATCTATAGTTTGGGATCTGGGGTCCCATCAAGGTTCAGAGGCACTAGATCTGGGACA

GATTACACTCTCACCATCAGTAGCCTGCAGCCTGAAGATGTTGCTACTTATTACTGT

> VK1-178

;

;

GGCATCCAGTTGACACAGCCTCCATCTGCA---TCTGCATCTGTGGGAGACACAGTCAAG

ATCAGTTGCCAGGCCAGTTTAAACTGGTATCAGCAGAAACCAGGGCAAGCTCCTAAACGC

CTGATCTATAATTTGGAGTCTGGAGTCCCATCAAGGTTCAGTGGCAGTGGATCTGGTACA

GGTTTCACTCTCACCATCAGCAGCCTGCAGCCTGAAGACTTTGCAACTTACTACTGT

> VK1-174

;

;

GACATCCAGATTACTCAGACTCCATCCTCCTTGTCTGCATCTGTGGGAGACACAGTCACC

ATCAATTGTCGGGCCAGTTTAAACTGGTATCAACAGAAACCAGGACAACCTCCTAAACGC

CTGATCTACAGTTTGGAATCTGGGGTCCCATCGAGGTTCAGAGGTAGTGGATCTAGGACA

GATTACACTCTCACCATCAGTAGCCTGCAGCCTGAAGATGTTGCTATTTATTACTGT

> VK3-170

;

;

CAAACCCTGTTGACCCAGACTCCAGCCTCCTTGGCTGTGTCTCCAGGAGAAACGGCCACG

GTCAATTGCAGGGCCAGTCTAGCATGGTACCAGCAGAAACCTGGGCAGGCTCCCAAGCTT

CTCATCTATAGCAGGGCCACTGGTGTCCCAGCCCGGTTCAGTAGCAGCGGGTCAGGGACA

GACTTCACTCTCACCATCAGCAGCCTGCAGCCTGAGGATGTTGCTGTTTATCACTGT

> VK6-164

;

;

AGCGTTGCCCTGACACAGTCCCCAGCCTTCCTGAGTGTGACTCCAGGTGAGAGTGTCTCC

ATCAGCTGCAGGGCCAGTTTAAACTGGTATCAGAAGAAACCAGGCGAGTCTCCTAAAATT

CTCATTAATAACCGATATGCCGGTGTCTCTGATCGCTTCATTGGCATATACAGTGGGACA

GAATTCACTTTTAAAATCAGCAGGGTAGAAGCTGGAGACGCTGGCACCTATTACTGT

> VK1-159

;

;

GACATCCAGTTAACACAGCCTCCATCCAGA---GCTGCATCTGTGGGAGACACAGTCACC

ATCAGTTGCAGGGCCAGTTTACACTGGTATCAGCAGAAACCAAGGCAGGCTCCTAAACCC

CTGATCTATAATTTGCAATCTGGAGTCCCAACAAGAGTCAGTGGCAGTGGATCTGGGACA

GATTTCACTCTCACCATCAGCAGCCTGCAGCCTGAAGACTTTGCAACTTACTACTGT

> VK3-155

;

;

CAAACCCTTCTGACACAGACCCCAGCATTCCTGGCTGTGTCTCCTGAAAAATCATTGACT

CTCATATGCAGGGCCAGTCTAGAATGGTACCAGCAGAAACCTGGGCAGGCTCCTAGGCTC

CTCATCTATAGCAGGGCCATTAGGGTTCCATCCCCATTCAAAGGCAGTGTGTCAGGTACA

GACTTCATTCTCACCATCAGCAGCCTGCAGCCTGAGGATGTTGCAGTTTATCACTTT

> VK1-153

;

;

GACATCCAGATGACTCAGACTCTCTCCTCCTTGCCGGCATCTGTGGGAGACACAGTCACC

ATCAAGTGTAAGGCCAGTTTAAATTGGTACCAACAGAAACCAGGGAAAGCTCCTCAACTG

CTGATCTATATTTTGGGATCTGGGGTCCCATCAAGGTTCAGTGGCAGTGGATCTGGAACA

GATTACACTCTGACCATCAATAGCCTGCAGTCTGTAGATGTTGCCACTTATTACTGT

> VK1-152

;

;

GATATCCAGTTGACACAGCCTCCATCTGCA---TCTGCATCTGTGGGAGACACTGTCAAG

ATCAGTTGCCAGGCCAGTTTAAAATGGTATCAGCAGAAACCAGGGCAAACTCCTAAACTC

CTGATCTATTATTTGCAGTCTGGGGTCCCTTCAAGGTTCAGTGGCAGTGGATCTGGGACA

GATTTCACCCTCACCATCAGTAGCCTGCAGCCTGAAGACTCTGCAACTTATTACTGT

> VK1-147

;

;

GATATCCAGTTGACACAGCCTGCATCCACA---TCTGCATCTGTGGGAGACACCATCACC

ATCAGTTGCCAGGCCAGTTTAAGCTGGTATCAGCAGAAACCAGGGCAAGCTACTAAACTC

CTGATCTATAATTTGTACTCTGGTGTCCCATCAAGGTTCAGTGGCAGTGGATCTGGGACA

GATTTCACTCTCACCATCAGTAGCCTTCAGCCTGAAGACTTTGCAATTTATTACTGT

> VK1-145

;

;

GACACCCAGATGACCCAGTCTCCATTCTCGCTGTCTGTGTCTCCTAGAGACAGAGTCACC

ATCACTTGCAGAGCCAGTTTAAATTGGTATCAGCAGGAACCAGGGAAAGCTCCTAAACTC

CTGATCTATTTTCTGCAACCTGGGGCCCTACCCAGAATCTGTGGCAGTGGATCTAGAGCA

GATTACACAATCACCATCAGCAGCCTGGAGCCTGAGGATGTTGCAGTTTACTGCTGT

> VK1-144

;

;

GACATCCAGATGACCCAGTCTCAATCTTCCCTGTCTGCCTCACTTGGAGACAGAGTCGCC

ATCACTTGCAAAGCAAGTTTGGCCTGGTATCAGCAGAAACAAGGGAAAGCTCCTAAGCTC

CTCATCTATAATTTGGCTTCCGGGGTCCCATCCAGGTTCAGTGGCAGTGGATCTGGGACA

GATTACAGTCTCACAATCAGTTCTGTGGAAGCTGAAGATATTGCAACTTATTACTGT

> VK1-142

;

;

GACATCCAGATGACCCAGTCTCCATCTTCCATATCAGCATCTTTAGGTGACATAGTCACC

ATCAGTTGTCATGTGAGTTTATTCTGGTATCAGCAAAATCCAGGGAAAGCTCCTAAATTG

CTGATCTATAACTTGCACACTGGGGTCCCACAGAGGTTCTTTGGCAGTGGGACTGCAACA

GATTTCACTCTCACCATCAGCAGCCTGCAGCCTGAGGACATTGCAACTTATTACTGT

> VK1-140

;

;

GACATTCAGATGACCCAGTCTCCATCCTTCCTCTCTGCATCCCTTGGAGACAGAGTCACC

ATTACTTGCAAAGCCAGTTTGGCCTGGTATCATCAGAAACCAGGGAAAGCTCCTAGGCTC

CTCATTTATAATTTGGATTCCGGGGTCCCATCCAGGTTCAGTGGCAGTGGTTCTAGGACA

GATTACACTTTCACTATCAGTGCTGTGGAGCCTGATGATGTTGGAATTTACCACTGT

> VK1-131

;

;

GACATCCAGTTGACCCAGTCTCCCT---CTGTGTCTGCATCTTTAGGAGAGAAAGTCACC

ATGACTTGCAAAGTCAGCTTGCACTGGTATCAGCAGAAACCTGCCCAAACACCAAAACTG

CTAATCAATACTTTGCAGTCAGGTATCCCCTCCAGGTTCAGTGCCAGTCGATCTGGAACA

GATTTCACTCTCACCATCAGCAACCTGGAACCTGAAGATGTTGCATCTTATTTTTGT

> VK1-126

;

;

GACATCCAGTTGACCCAGACTCCAGCTTCACTTTCTTCAGCTTTAGGAGACACAGTCATC

ATCACTTGCAGGGCTAGTTTAAATTGGTATCAGCAGAAACCAGGAAAAGCTCCTAAACTC

CTGATCTATAGTTTGGAATCTGGGGTCCCTTCAAGGTTCAGTGGCAGTGGATATGAGACA

GATTTCACTCTCACCATCAGCAGCCTGCAGCCTGAAGACATTGCAACTTATTACTGT

> VK2-124

;

;

GAAGTTGTGCTGACCCAGACTCCACTCTCCCTGTCCATCACCCCTGGAGAGTCGGCCTCC

ATCTCCTGTAGATCCAGCTTGTATTGGTACCTGCAGAAGCCAGGCCAGGCTCCAAAGCTC

CTGATTTACAACAGGGTTACTGGGGTCTCAGACAGATTCAGAGGCAGTGGGTCAGGGACA

GATTTCACCCTGCAAATCAGCAGCATGCAGTCAGAAGATACTGGGGTTTATTACTGT

> VK1-117

;

;

GATATCCAGTTGACCCAGTCTTCCT---CTGTGTCTGTGTCATTAGGAGAGAAAGTTACC

ATCACCTGTCTAACCAGCTTACACCGGTATCAGCAGAAAATTGGGAAAGCTCCTAAACTT

CTCATCTATAGTTTGGAGTCTGTGGTCCCTTCAAGGTCCAAAGGCAGTGGGTATGGGAGA

GATTTCACTCTCACCATCAACAGCCTGGAGCCTGAAGATGCTGTGTCTTTTTACTGT

> VK1-115

;

;

GACATCCAGTTGACCCAGTCTCCCT---CTGTGTCTGCGTCTTTAGGAGAGAAAGTCACC

ATGACTTGCAAAGCCAGCTTACACTGGTATCAGCAGAAACCTGGCCAAACTCCTAAACTC

TTAATCCGTACTTTGCAGTCTGGTATCCCCTCCAGGTTCAGTGGCAGTGGATCTGGGACA

GATTTCACTCTTGCCATCAGCAGCCTGCAGCCTGAAGATGTTGCGACCTATTACTGT

> VK1-108

;

;

GTCATCCAGATGATGCAGTATTCATCATCCCTGTCTGTGTCTGTTGGAGTCAAGGTCAGT

TTCAGGTGCCAGGCTAGTTTAGACTGGTATCAGCAAAAAGCAGGAAAGACGCCTACACTC

CTGATCTATAGCTTACACTCTGCTGTCCCATCAAGATTTAGGGGCAGTGGGTCTGGGACA

GATTTCACTCTCACCATCAGCAGCCTGCAGCCTGATGATTTTGCAAATTATTCCTGT

>VK5-105

;

;

CAAATTGTGCTTACCCAGTCTCCAGCATCCATGGCTGCTTCTCCAGGGGAAAAGGTCACC

ATCACCTGCAAAGCTAGCTTGCACTGGTACCAACAAAAGCCAGGATCCTCTCCCAAGCTC

CTAATTTATAAACTGGCCTCGGGAGTCCCAGCTCGCTTCAGTGGGAGTGGGTCAGGGACT

TCTTACTCGCTCACAATCAGCAACGTGGAGGCTGAAGATTTTGCAACCTATTACTGT

> VK1-99

;

;

GAAATTCAGATGACACAAACTTCCTTCTCCATATCTGTATCTGTAGGAGAGAGAGTCATG

ATCAATTGGAGGGTGGGTTTAGATTGGTATCAAAAGAAACCAAGGAAAGCTCCAAAATGT

TTAATCTACAATTTGACATCTAGTGTCCCATCAAGGTTCTGTGACAGTGGATCTGGGACA

AATTTCACTCTCATCATCATCAGCCTGCAGCCTGATGATGTTGCAACTTATTACTCT

> VK4-83

;

;

GACATTGTGATGACCCAGTCTCCAGCTTCCCTGGCAGTGTCTCCTGGAGAGAGCGCCACC

ATCCACTGCAAGTCCAGCTTAGTCTGGCTCCAGCAGAAACCAGGGCAATCTCCCAAACAG

CTCATCTACACCAGAGAAACTGGGGTCCCAGACCGGTTCAGTGGCAGTGGGTCTGGCACA

GATTTCACTCTCACCATCAGCAGGGTGCAGGCTGAAGATGTGGCAGATTATTACTGT

> VK4-81

;

;

GACATTGTGATGACCCAGTCTCCAGCCTCCCTGGCAGTGACTCCTGGAGAGAGTGCCACC

ATCCACTGCAAGTCCAGCTTAATCTGGTACCAGCAGAAACCAGGGCAATCTCCCAAACTG

CTCATCTACACCCGAAATACTGGGGTCTCAGACCGGTTCAGTGGCAGTGGGTCTGGCACA

GACTTTACTCTCACCATCAGCAGGGTGCAGGCTGAAGATGTGGCAGATTATTATTGT

> VK4-77

;

;

GAAATTGTGATGACCCAGTCTCCAGCCACCCTGGCTGTGTCTCCTGGAGAGAGAGTCACC

ATCCACTGCAAGTCAAGCTTAAACTGGTACCAGCAGAAACCAGGGCAATCCCCCAAACTG

CTCATCCACACCCGAGCATCTGGTGTCCCAGACCGGTTCAGTGGCAGTGGGTCTGGCACT

GATTTTACTCTCACCATCAGCAGCTTGCAGGCTGAAGATGTGGCAGATTATTTCTGC

> VK4-76

;

;

GACCTTGTTATGACACAGTCTCCAGCCTCCCTTACAGTGTCACCTGGAGAGAGCAACACC

ATCCACGGCAAGTTCAGCTTATCATGGTACCAGCATAAATCAGGACAAACTCCCAAACTT

CTCATCTACAGCAAGGAATCTGGGGTCCCAGACAGGTTCAGTGGCAATAGGTCTGTTACA

AATTTCATTCTCACCAATACCAGGATTCAAGGTGAAGATATGGTGGATTATTACTGT

> VK4-74

;

;

GACATTGTGATGACCCAGTCACCAGCCTCCCTGGCAGTTTCTGCTGGAGAGAGTGCCACC

ATCCGCTGCAAGTCCAACTTAAGCTGGTACCAGCAGAAACAAGGACAACCTCCTAAACTG

CTCATCTACACCCGGGTATCTGGGGTCCCAGATAGTTTCAGTGGCAGTGGATCTGGCATG

CATTTCACTCTCACCATCAGCAGGATGCAGGATAAAGACTTTGCAGAATATTACTGC

> VK4-70

;

;

GACATTGTGATGACCCAGTCTCCAGGCTTTCTGGCAGTGTCTGCTGGAGAGAGCATCACC

ATTCACTGCAAGTCCAGCTTATCCTGGTACCAGCAGAAACCAGGACAATCTCCCAAACTG

CTCATCTACTCCCGGGTATCTGGAATCCCAGACCGGTTCAGTGGCAGTGGTTCTGGCATG

GATTTCACTCTCACCATCAGCAATGTGCAGGCTGAAGATGTGGCATATTATTACTGC

> VK4-69

;

;

GACAATGTGGTGATCCAGTCTCCAGGCTCCCTCTTGCTGTCTGTTGGAGAGAGTGCCACC

ATCCACTGCAAATCCAGCTTATCCTGGTACCAGCAGAAACCAGGACAATCTCCCAAACTG

CTCATCTACACTCGGGCATCTGGGGTCCAAGATCGGTTCAGTGGCAGTGGGTCTGGCACG

GATTTCACTCTCACCATCAGCAAAGTGCAGGCTGAAGATGCTGGGAGTTATTACTGT

> VK1-65

;

;

GACATTCAGATGACCCAGTCTCCATCTTCTCATTCTGCATCTGTAGGAGACGTTGTTACC

ATCAATTGCAAGGCCAGTTTAGCCTGGTATCAACAGAAACCAGGGAAAGAGCCTAAATTT

CTGATATATAGATTAGCATCTGGGATCCCCTCCAGGTTCAGTGGCAGTGGATCTGGGACA

GACTTCACTCTCACCATCAGCAGCCTGCAGCCTGAAGATGTTCTAACTTATTTCTGT

> VK4-48

;

;

GACATTGTGATGACCCAGTCTCCAGCCTCCCTGGCAGTGACTCCTGGAGAGAGAGCCACC

ATCCACTGCAAGTCCAGCTTAGACTGGTACCAGCAGAAACCAGGGCAATCTCCCAAACTA

CTCATCTACACCCGAGAAACTGGGGTCCCAGACCGGTTCAGTGGCAGTGGGTCTGGCACA

GACTTTACTCTCACCATCAGCCCAGTGCAGGCTGAAGATGTGGCAGATTATTACTGT

> VK4-41

;

;

GACATTGTGATGACCCAGTCTCCAGCCTCCCTGGCTGTGTCTCCTGGAGAGAGTACTACC

GTCCACTGCAAGTCCAGCTTCAGTTGGTACCAGCAGAAACCAGGCCAATCCCCCAAACCG

CTCTTGTACACTTGGCAATCTGGGGTCCCAGATTGTTTCAGTGGCAGTGGGTCTGGCACC

GATTTCACTCTCACCATCAGTAGCCTGCAGGCTGAAGATGTGGCAGATTATTACTGC

> VK4-39

;

;

GACATTGTGATGACCCAGTCTCCAGCCTCCCTGGCAGTGTCTCCTGGAGAAAGTGCTACC

ATCCACTGCAAGTCTAGCTTATCCTGGTACCAGCAGAAACTAGGGCAAACTCCCAAACTG

CTCATCTACACCCGAGAAACTGGGATCCCAGAGCGGTTCAGCGGCAGTGGGTCTGGCACA

GATTTCACTCTCACCATCAGCAGCCTGCAGGCTGAAGATGTGGCAGATTATTACTGC

> VK4-37

;

;

GACATTTTGATGACCCAGTCTCCAGCCACACTGGCTGTGTCTCCTGGAGAGAGAGTCACC

ATCCACTGCAAGTCCAGCTTAAGCTGGTACCAGCAGAAACCAGGCCAATCCCTGAAACGG

CTCATCTACACCCGGGCATCTGGGGTCCCAGACCGGTTCAGTGGCAGTGGGTCTGGCACC

GATTTCACTCTCACCATCAGCAGCCTGCAGGCTGAAGATGTGGCAGATTATTTCTGC

> VK4-36

;

;

GACATTGTTATGACCCAGTCTACAGCCTCCCTGACAGGGCCAGCTAGAGAGAGCACCACC

ATCTACTGCAAGTTCAGCTTATCATGGTACCAGCAAAAACCAGGAGAACCTCCCAGACTA

CACATCTACAGCAAGGAATCTGGTGTCCCAGACAGGTTCAGTGGCAGTGGGCCTGGCATG

GATTTCACTCTCACCATTACCAGCATTCAAGGTGAAGATGTGGTGGATTATTACTGT

> VK2-22

;

;

GATGTTTTGATGACCCAGACCCCACTCTCTCTAATCATTGCCCCTGGAGAGCCAGCCTCC

ATGTCCTGCAGGTCCAGTTTGAGTTGGGTGGTGCATAAGCCAGAACAGGCTCCACAAGGA

ATAATATATAAACATGAATCTTGGACCCCAGAGAGGTTCAGTGGCAGTGGGACAGGGACA

GATTTCACACTCGACATTAGCACGGTGGAGGCTGAGGACGCTGGAGTTTATTACTGT

> VK2-20

;

;

GATATTGTGATGACACAGACCCCACTCTCTCTCTCCGTTTCCCCTGGAGAGCCAGCCAGC

CTCTCCTGCAAGGCCAGTTTAAACTGGGTTGTGCACAAGCCAGGCCAGGCTCCTAGGGGC

ATGATTTATAACCGGTATTCTGGCATCTCAGAGAGGTTCAGTGGCAGTGGGTCAGGGACA

GATTTCACACTCAAAATCACCAGGGTGGAGCCTGAGGATGCTGGAGTTTATTACTGT

> VK2-16

;

;

GATGTAGTGATGACCCAGACTCCACTCTTGCTATCTGTCTCCCCTGGAGAGCCAGCCTCC

ATCTCCTGCAGGTCCAGTTTGCACTGGTTGGTGCACAAGCCTGGCCAGGCTCCAAGGGGT

GTGATTTATGACAAGTACTCTTGGACCCCAGAGAGGTTCACTGGCAGTGGGTCAGGAACA

GATTTCACACTCAAAATCAGCAGGGTGGAGGCTGAGGATGCTGGAGTTTATTATTGT

> VK2-15

;

;

GATGTTCTGATGACCCAGACCCCACACTCTTTGTCCGTCACCCCTGGAGAGCCGGCCACT

ATCTCCTGCAGGTCCAGTTTTAATTGGCTTGTGCAGAAGCCAGGCCAGGTTCCAAGGCTC

ATCATTTATAAAAGAGACTCTTGGGTCTCTGACAGGTTCAGTGGCAGTGGGTCATGGACA

GATTTCACACTCAAAATCAGCAGGGTGGAGGCTGAGAATGCTGGAGTTTATTACTGT

> VK2-14

;

;

GATGTTGTATTGACCCAAACCCCACTATCCCTCTCTGTCACCCCTGGAGAGCCAGCCTCC

ATCTCCTGCAGGGCCAGTTTGCACTGGGTGGTGCACAAATCAGGCCAGGATCCACAAAGA

ATGATTTATTATCAGAAATCTTGGGTCTCTGACAGGTTCAGTGGCAGTGGGTCATGGACA

AATTTCATTCTCAAAATCAGCAGGGTGGAGGCTGAGGGCGCTGGAGTTTATTACAGT

> VK2-11

;

;

GATGTTGTGGTGACCCAGACCCCACTCTCTCTGTCCATCGCCCCTGGAGAGCCGGCCTCT

ATCTCCTGCAGGTCCAGCTTGCATTGGTTTGTGCACAAGCCAGGCCAGGCTCCACATGGT

GTGATTTACTACCGGTATTCTGGTATCTCAGACAGATTCAGTGGCAGTGGGTCAGGGACA

GATTTTGTCCTCAAAATTAGCAAGGTGGAGGCTGAGTACGCTGGAGTTTATTACTAT

> VK2-10

;

;

GACATAGTGATGACCCAGACCCCACTCTCTCTGTCTGTCACCCCTGGAGAGCCGGCCGTT

ATCTCCTGCAGGACCAGTTTTTACTGGTTGGTGCACAAACCAGATCAGGTTCCAAGGGGT

GTGATTTATAAAAAGTTCTCCTGGACCCCAGACAGGTTCAGTGGCGGTGGCTCAGGGACA

GATTTCACACTGATAATCAGCAGGGTGGAGGCTGAGGATGCTGGAGTTTATTACTGT

> VK2-7

;

;

GATGTTGTGATGACCCAGACCGCACGCTCTCTGTCCATCGCCCCTGGAGAGCCAGCCTCC

ATCTCCTGCAGGGCCAGTTTGAGTTGGCTTGTGCAGAAGCCAGGCCAGGCTCCTCGGCTC

ATGATTTATAATCGGGAATCCTGGGTCCCTGACAGGTTCAGTGGCAGTGGGTCAGGGACA

GATTTCACTCTCAAAATCAGCAGGCTGGAGGCTGAAGACGCTGGAGTTTATTACTGT

> VK2-3

;

;

GATGTTGTGATGACCCAGACCCCACTCTCTCTGTCCGTCTCCCCTGGAGAGCCGGCCTCC

ATCTCCTGCAGGGCCAGTTTGCATTGGGTGGTGCACAAGCCAGGCCAGGCTCCACGAGGA

ATGATTTATAATAAATACTCTTGGACCCCAGAGAGGTTCAGTGGCAGTGGGTCAGGAACA

GATTTCACACTCAAAATCAGCAGAGTGGAGGCTGAGGATGCTGGGGTTTATTACTGT

> VK2-2

;

;

GATGTTTTGTTGTCCCAGACCCCACTCTCTCTGTCTATCACACCTGGAGAGCCGGCCTCC

ATCTCCTGCAAGTCCAGTTTGCATTGGGTTGTCCACAAACCAGGCCAGGCTCCACGAGGA

ATGATTTATAATCGGGAATCTGGGACTCCAGAGAGATTCAGTGGCAGTGGGTCAGGGACA

GATTTCACACTCAAAATCAGCAAAGTGGAACCAGAGGATTCTGGAATTTATTACTGT

> VK2-1-490

;

;

GATGTAGTGATGACCCAGACTCCGCTCTCCCTATCTGTCTCCCCTGGAGAGCCAGCCTCC

ATCTCCTGCAGGTCCAGTTTGCACTGGTTGGTGCACAAGCCTGGCCAGCCTCCAAGGGGT

GTGATTTATGAAAAGTACTCTTGGACCCCAGAGAGGTTCACTGGCAGTGGGTCAGGAACA

GATTTCACACTCAAAATCAGCAGGGTGGAGGCTGAGGATGCTGGAGTTTATTATTGT

> VK2-4-165

;

;

GATGTTGTGATGACCCAGACCCCACTCTCTCTGTCCATCACCCCTGGAGAGCCGGCCTCT

ATCTCCTGCAGGTCCAGCTTGCATTGGGTTGTGCACAAGCCAGGCCAGGCTCCACATGGT

GTGATTTACTACCGGTATTCTGGTATCTCAGACAGATTCAGTGGCAGTGGGTCAGGGACA

GATTTTGTCCTCAAAATTAGCAAGGTGGAGGCTGAGGACGCTGGAGTTTATTACTGT
