# Supplementary material for: Immunoglobulin Genomics in the Guinea Pig (Cavia porcellus)
Source: PLoS One. 2012 Jun 22;7(6):e39298. doi: 10.1371/journal.pone.0039298 (PMC3382241; doi:10.1371/journal.pone.0039298)
Supplement: Figure S3 — Multiple sequence alignments of guinea pig Vλ genes. (DOC) [file pone.0039298.s003.doc]

Figure S3

> VL12-8

;

;

---TCCTATGTGCTCACACAGCCACCTTCAGTGTCGGTGTCCCTGGGACAGACAGCCTCA

ATTACCTGCTCTGGAAAC----GAACTGCCAA-----GTAGATATGCATGTTGGTTCCAG

CAGAAGCCAGGGCAGCCTCCTGTGCAAGTAATATA-CAAAGACAGTGAGTGGCCCTCAGG

---------------GATCTCTGAGAGATTGTCTGGTTC------CAACTCAGGGACCAC

AGCCACCCTGACCATCAACGGTGTCCAGGCTGAAGATGAAGCTGATTATTATTGT-----

----

> VL12-6

;

;

---TCCTATGTATTCACACAGCCACCTTCAGTGTCGGTGTCCCCGGGACAGACAGCCACA

ATTACCTGCTCCGGAGAT----TCACTGCCAA-----GTAGATATGCACACTGGTACCAG

CAGAAGCCAGGACAGACTCCTGTGAATGTAATACG-CAAAGACAGTGAACGGCCCTCAGG

---------------GATCTCAGAGAGATTCTCTGGCTC------CAGGTCAGGGACCAC

AGCCACCCTGACCATCAGCAGAGTCCAAGCTGAAGATGAGGCTGATTACTACTGT-----

----

> VL3-4

;

;

---TCCTATGTGCTGACACAGCCATCTTCCATGTCAGTGTCTCCTGGAGAGACAGCCAGG

CTGACCTGTGAGGGAAACA---ACATTGGAGG------TAAAGCTGTGCACTGGTACCAG

CAGAAGCCAGCCCAGGCCCCCATGCTGGTCATGTA-TTATGATAATGAACGGCCCTCGGG

---------------GATTCCCGACCAATTCTCCGGTGC------CAACTCGGGCAACAC

AGCCACCCTGACCATCACTGGCGCTCAGGCTGAGGATGAAGCCGACTATTACTGT-----

----

> VL9-39

;

;

---CAGCCTGTAGTGACTCAACCACCCTACCTCTCTGTATCTTCTGGAGCAAACGCCAGA

CTCACCTGTTCTCTGAGCAGTGGCTCTCCACTTTCTGACTACCACATTTCCTGGTACCAG

CAAAAGACAGGGAACCGTCCTCAATTTTTACTGAGATACTACTCAGATTCAAATAAACAG

----CAAGGCTCTGGGGTTCCCAACCGCTTTGTTGGATCCAAAGATCCCTCAGCCAATGC

AGGAATTTTGTTCATCTATGGACTTCAGTATGAGGATGAGGCTGACTATTACTGT-----

----

> VL4-138

;

;

---CAGCCTGTGCTGAAACAGTCACCCTCTGCTTCTGCTTCTCTGGGAGCCTCAGTCAAG

CTCACCTGCACTTTGAGTAGTGAGCACAGCAG------TTACTTCATTTCGTGGTATCAA

CAGCAGCCAGGGAAGTCCCCTGGTTATGTGATGAG-TGTTTACAGTGATGGAAGACATAG

---CACGGGGGATGGGATACCTGATCGATTCACAGGATC------CAGCCCTGGGGCTGA

TCGCTACTTAACCATCTCCAACATCCAGCCTGAAGACGAAGCTGACTATTTCTGT-----

----

> VL9-134

;

;

---CAGCCGGTGCTCACTCAACCACCCTTCCTGTCCGCGTCTTCTGGAGCAAGTGCCGTA

CTTACCTGCACCCTGAGCAGCGGCTTTGTAGTTTCTGGTTACCACATTTCCTGGTACCAG

CAAAAGTCAGGGAACCATCCTCGATATCTACTGAGATATTACTCAGACTCAAATCAGCAC

----CAGGGCTCTGGGGTTCCTAGCCGCTTCACTGGATCCAAAAACCCCTCAGCTAACGC

AGGAATTTTGCTCATCTCTGAACTTCAGTATGAGGATGAGGCTGACTACTACTGT-----

----

> VL6-132

;

;

TCTCAGGCTGTGGTGACTCAGGAATCTTCACTCTCTGTGACTCCTGGAGGGACAGTCACC

CTCACTTGTGGATTTAGCACTGGGGCTGTCACAACCAGTAACTATGCTAAATGGGTCCAA

CAGAATCCTTACCAAATACTCCAGGGTCTAATACG-TGATACCAGCAATCAGGTCCCAGG

---------------AGTTGCTGCCAGATTCACAGGCTC------TCTCCTTGGAAACAA

AGCATCTCTCACCATCACAGGGGCCCAGCCTGAAGATGAGGCCACCTATTACTGT-----

----

> VL7-129

;

;

TCTCAGACTTCAGTCACCCAGGAGCCATCAATGTCTATGTCTTCAGGAGAGACAGTCACT

CTCACCTGTTGCCTAAGCTCTGGGTTCGTCTCTACTAATAACTATCCTAGCTGGTACCAG

CAGAACCTTGGCCAGGCTCCATATGCTCTTACCTA-CAACACAAACAGCCATTTCTCTGG

---------------AGTTCCTGATC-A-----------------CTTGTCTGGATCCA-

--TTGCCCTTACCATCATGATAGCTCAAACAGAGGATGAAGCTGATTATTACTGT-----

----

> VL1-125

;

;

---CAGGCAGTGCTGAGTCAGCCGCCCTCAGTGTCTGGGCCTCTAGAAGGGACGGTCACC

ATCTACTGCACTGGAAGCAGCACCAATTTGGGTTCTGGCTATTATGCCCACTGGTACCAG

CAGCTCTCAGGAATGTCTCCCAAACTCATCATTTA-TGAAAATAGCAATCGACCCTCAGG

---------------GATCTCTGGTCGATACTCTGGCTC------CAAATCTGGCAACTC

AGCCTCCCTGACTATCAGTGGGCTCCAACCTGAGGATGAGGCTGATTATTACTGT-----

----

> VL5-122

;

;

---CAGACTGTGGTGACCCAGGTATTCTCACTCTCTGCATGTCCTGGAGCAGCTGCCAGA

CTTTATTGCACCTTGAGAAGTGACATCAGTGTCAGTGGTGCGCACACGTACTGGTATGAG

CAGAAGCCACAGAGTCCTCCCAGGTTTCTCCTACACTACTACTCAGACTCCAATGAGAAA

----GGGGGGTCTGACATCCCCAGTCACTTGTTTGGTTCAAAAGACATCTCAGCCAATGT

AGGCATTTTGCATATTTCTGGGCTGCAGCCTGAGGATGAAGCTGACTATCACTGT-----

----

> VL4-121

;

;

---CAGCTTGTGCTGACTCAGTCACCTTCTGCCTCTGCATCCCTCGGAGCCTCGGCCAAG

CTCACCTGCACCCTCAACAGTGAATATAAACA------CTATGGCATTGCATGGTTTCAG

CAGTACCCGGGGAAGGCTCCTCAGTATTTGATGTG-GGTTAAGAGCGATGGAAGCCTCAT

---TAAAGGAAATGGAATTCCTGACCGCTTCTCAGGTTC------CAGCTCTGGGACCGA

ACGCTACTTGACCATCACCAACATCAATTCTGCGGATGAAGCTGACTATATCTGT-----

----

> VL6-116

;

;

TCCCAGGCTGTGGTGACTCAGGAATCTTCACTGTCCATCTCTCCTGGAGGGACAGTCACA

CTCACCTGTGCTTCCAGTACTGGGGCTGTCACAACTAGTAACTATGCTGCCTGGGTCCAA

CAAAAACCCTCTGAAATAACAAAGAGTCTAATATA-TGGTACCAGCAACCGAAGCCCAGG

---------------GGTTCCTGCCCGATTCACAGGCTC------CTTCCTTGGAAACAA

GGCTGCCTTCACAATCACAGGGGCCCAGACTGAAGATGAGGCCACCTATTACTGT-----

----

> VL6-115

;

;

---AAGGCTGTGGTGACTCAGGAATCTTCACTGTCCATCTCTCCTGGAGGAACAGTCACA

CTCACCTGTGCTTCCAGTACTGGGGCTGTCACAACCAGTAACTATGCTACCTGGGTCCAA

CAAAAACCCTCTGAAACATCAAAGGGGCTAATATA-TGGTACCAGCACCCGAAACCCGGG

---------------GATTCCTGCCCGATTCACAGGCTC------CTTACTTGGAGACAA

GGCTGCCTTCACCATCACAGGGGCCCAGACTGAAGATGAGGCCACCTATTCCTGT-----

----

> VL1-109

;

;

---CAGGCAGTGCTGACTCAGCCGCCCTCAGTATCTGGACCTCTAGGAGAGACAGTCACC

ATCTCCTGCACTGGAAGCAGCACCAATATTGGTTATGGCTATACTGCCAGCTGGTACCAG

CAGCTCTCAGGAATGACTCCCAAACTCATCATTTA-TAGAAACAGCAATCGACCCTCTGG

---------------GGTCTCTGATCGATACTCTGGCTC------CAAATCTGGCAACTC

AGCCTCCCTAACCATCAGTCGGCTCCAACCTGAGGATGAGGGTGATTATTACTGT-----

----

> VL5-106

;

;

---CAGAATGTAGCGACCCAGGTATCCTCACTCTCTTCATCTCCTGGAGCAGCTGTCAGA

CTTACTTGCACCTTGAGAAGTGACATCAGTGTCAGTGGTGTGCACATGTACTGGTACCAG

CAGAAGCCACAGAGCCCTCCTTGGTTTCTGCTACACTACTACTCAGACTCCAATAAGAAA

----CAGGGGTCTGACATCCCCCGCCGCTTCTCTGGCTCAAAAGACGCCTCAGCCAATGC

AGGCATTTTGCATATTTCTGGGCTGCAGCCTGAGGATGAAGCTGACTATCACTGT-----

----

> VL8-104

;

;

---CAGCCTGTGCTGACTCAGCTGCCCTCTGCATCTGCCTCCCTGGGACAGACACACAAA

CTCACCTGTACCCTGAGCAGTGGCTACAGTAA------CTACAATGTGGACTGGTACCAG

CAGAGCCAGGGAAAGAGCCCTCGGTTTGTGATGCG-AGTGGGTACTAGTGGTATTGTGGG

ATCCAAGGGGAGTGGAATCCCTGATCGATTCTCAGGATC------AGGTTCTGGCTCTGA

ACGGTACCTGACCATCCAGAACATCCAAGAAGAAGATGAGAATGTCTACTACTGT-----

----

> VL6-102

;

;

TCCCTGGCTGTGGTAACTCAGGAATCTTCACTGTATGTCTCTCCTGGAGGAACAGTCACA

TTCACCTGTGCTTCTAGTACTGGGGCTGTCACAACCAGTAACTATGCTGCCTGGCTCCAA

CAAAAACCCTATGAAACACCACAGGGGCTAATAGA-TGATACCAGCCACCGGATCCCTGG

---------------TGTCCCGGCCCGATTCACAGGCTC------TTTGCTTGGAGACAA

GGCTGCTTTCACCATCACAGGGGCTCAGACTGAAGATGAGGCCACCTATTACTGT-----

----

>VL5-99

;

;

---CAGGATCTGGTAACTCAGGAACCTTCACTGTCTGCATCTCCTGGAGCAGCTGCCAGA

CTTACTTGTACCTTGAGAAGTGACATCAGTGTTGGGGGAAAAAACTTGTACTGGTACCAG

CAGAAGCCAGGGAGCCCTCCCAGGTTTCTCCTATACTACTACTCAGACTCAGATAAGCAA

----CTGGGTTCTGGCTTCCCCAACCGCTTCTCTGGTTCAAAAGACACTTCAGCCAATGC

AGGCATTTTGCATATTTCTGGGCTGCAGCCTGAGGATGAGGCTGACTATTACTGT-----

----

> VL6-98

;

;

TCCCAGGCTGTGGTGACTCAGGAATCTTCACTGTACATCTCTCCTGGAGGGACAGTCACA

CTCACCTGTGCTTCCAGTACTGGGGCTGTCACAACCAGTAACTATGCTGCCTGGCTCCAA

CAGAAACCCTATGAAACACCCAAGGGGATAGTAGG-TAATACCAGCAACCGGATCTCTGG

---------------TGTTCCTGCCCGATTCACAGGCTC------CTTGCTTGGAGCCAA

GGCCTCCCTCACCATCACAGGAGCCCAGAATGAAGATGAGGCCACCTATTACTGT-----

----

>VL2-96

;

;

---CAGTCTGGCCTAAGTCAGGAAGCTTCATTGTCAGGGTCTGTGGGACAGAGGGTTACC

CTCTCCTGTACTGGAAGCAGCAGCAATGTTGG---AAGCTATGGTGCAGGCTGGTACCAA

AAGATTCCTGGTGCTGCTCCCAAAACTGTGATGCT-TGGAACTA---CTCGGCCTTCAGG

---------------GATCCCTGACCGCTTCTCTGGCTC------CAAGTCAGGAAACAG

AGCCACCCTGAGCATTTCGAATCTCCAACCCGAGGATGATGCTGATTATTACTGC-----

----

> VL1-95

;

;

---CAGGCAGTGCTGACTCAGCTGCCCTCAGTGTCTGGGGCTCAAGGAGAGATGGTCA--

-TCTCTTGCACTGGAAGCAGCACCAATTTTGGTTCTGGCTATGATGCCAGATGGTACCAG

CAGCTCTCAGGAAAAGCTCCAAAACTCATCATTTA-TGGAAATAGCAATCGACCCTCAGG

---------------GATCTCTGATCGATACTCTGGCTC------CAAATCTGGCAATTC

AGCCTCCCTGACCATTAGTCGGCTCCAACCTGAGGATGAGGCTGATTATTACTGT-----

----

> VL4-91

;

;

---CAGCTTGTGCTGACTCAGTCACCCTCTGCCTCTGCCTCCCTCAGAGCCTCAGCCAAG

CTCACCTGCACCCTCAATAGTGAATATGAAAC------CTATGGCATTGCATGGTTGCAG

CGGTACCTAGGGAAGGCCCCTAAGCATTTGATGTG-TATTAACAGTGAGGGAAACTTCAA

---TAAAGGAGATGGAATTCCTGATCGCTCCTCAGTTTC------CAGCTCTCGAACTGA

CCCCTGTTTGACCATCTCCAATATCAATTCTGGGGATGAAGCTGACTATATCTGT-----

----

> VL8-87

;

;

---CAGCCTGTGCTGACTCAGCTTCCCTCTGCATCTGCCTCCCTGGGACAGACACTCAAA

CTCACCTGTACCCTGAGCAGTGGCTACAGTAA------CTACAATGTGAACTGGTACCAG

CAGAGCCAGGGAAAGAGCCCTCGGTTTGTGATGCG-AGTGGGTACTAGTGGTATTGTGGG

ATCCAAGGGAGATGGAATCCCTGACCGATTCTCAGGATC------AGGCTCTGGCTTGGA

TCGTTACCTGACCATCCAGAACATCCAAGAAGAAGATGAGAATGTCTACTACTGT-----

----

> VL8-85

;

;

---CAGCCTGTGCTGACTCAGCTGCCCTCTGCATCTTTCTCCCTGGGACGGACACACAAA

CTCACCTGTATCCTGAGCAGTAACTACAGTAA------TTATACCATGGACTGGTTCCAG

CAGAGCCAGGGGAAGGGCCCCCGGTTTGTGAAGCG-AGTGGGTACTAGTGTAATTGTTGG

CTCCAATGGGCATGGAATCCCTGACCGATTATCAGGATC------AGGCTCTGGCTTAGA

TCGGTACCTGACTATCCAGAACATACAAGAAGAAGATGAGAACGTTTACTACTGT-----

----

> VL4-84

;

;

---CAGCCTGTGCTGAAACAGTCACCCTCTGCTTCTGCTTCTCCGGGAGGCTCGATCAAG

CTGACCTGCACTTTGAGTAGTGAGCACAGCAG------TTACTACATTGAATGGTATCAG

CAGCACCCAGGGAAGTCCCCTGGTTATGTGATGCA-ACTTAAGAGTGATGGAAGCCATAG

---CAAGGGGGACGGAATACCTGATCGATTCACAGGCTC------CAGCTCTGGGGCTGA

TCGCTACTTAACCATCTCCAACATCCAGCCTGAAGACGAGGCTGACTATATCTGT-----

----

> VL4-82

;

;

---CAGCCTGTGCTGAAACAGTCACCCTCTGCTTCTGCTTCTCCGGGAGGCTCGATCAAG

CTGACCTGCACTTTGAGTAGTGAGCACAGCAG------TTACTACATTGAATGGTATCAG

CAGCACCCAGGGAAGTCCCCTGGTTATGTGATGCA-ACTTAAGAGTGATGGAAGCCATAG

---CAAAGGGGACGGAATACCTGATCGATTCACAGGCTC------CAGCTCTGGGGCTGA

TCGCTACTTAACCATCTCCAACATCCAGCCTGAAGACGAGGCTGACTATTTCTGT-----

----

> VL4-80

;

;

---CAGGTTGTGCTGACTCAGTCACCCTCTGCTTCTGCCTCCCCTGGAGCCTCAGCCAAG

CTCACCTGCACCCTCAACAGTGAATATAAAAC------CTATGGAATTGCATGGTTTCAG

CAGTACCCGGGGAAGGCTCCTCAGTATTTGATGTG-GGTTAAGAGCGATGGAAGCTTCAA

---TAAAGGAGATGGAATTCCTGACCGCTTCTCAGGTTC------CAGCTCTGGGGCTGA

CCGCTACTTGACCATCACCAACATCAATTCTGGGGATGAAGCTGACTATATCTGT-----

----

> VL5-79

;

;

---CAGGATCTGGTGACTCAGGAACCCTCACTCTCTGCATCTCCTGGAGCAGCTGCCAGA

CTTACTTGCACCTTGAAAAGTGATATCAGTGTTGGTAATTACAGAATAAACTGGTTCCAG

CAGAAGCCACAGAGCCCTCCCCAGTTTCTCCTACACTACTACTCAGACTCAGATAAGCAA

----CTGGGTTCTGGCATCCCCAGCCGCTTCTCTGGTTCAAAAGACACCTCAGCCAATGC

AGGCGTTTTGCGTATTTCTGGGCTGCAGCCTGAGGATGAAGCTGACTATTACTGT-----

----

> VL5-78

;

;

---CAGGATCTGGTGACTCAGGAACCCTCACTTTCTGCATCTGCTGGAGCAGCTGCCAGA

CTTACTTGCACCTTGAGAAGTGACATCAGTGTGGGGGGAAGAAACCTGTACTGGTACCAA

CAGAAGCCAGGGAGCCCTCCCAGGTTTCTCCTATACTACTACTCAGGCTCAGATAAGCAA

----CTGGGTTCTGGCTTCCCCAGCCACTTTTCTGGTTCAAAAGACACCTCAGCCAATGC

TGGCATTTTGCATATTTCTGGGCTGCAGCCTGAGGATGAGGCTGACTATTACTGT-----

----

> VL5-74

;

;

---CAGGAACTGGTGACTCAGGAACCCTCACTCTCTGCACCTCCTGGAACAGCTGCCAGA

CTTACTTGCACCTTAAGAAGTGACCTCAGTGTTGGTAGTTACAGAATATTCTGGTACCAA

CAGAAGCCAGGGAGTCCTCCCAGGTTTCTCCTACACTACCACACAGATTCAGATAAGCAA

----CTGGGTTCTGGCTTCCCCAGTCGCTTCTCTGGTTCGAAAGACACCTCAGCCAATGC

AGGTGTTTTGCGTATTTCTGGTCTGCAGCCTGAGGATGAAGCTGACTATTACTGT-----

----

> VL6-71

;

;

TCCCAGGCTGTGGTGACTCAGGAACCTTCACTGTACATCTCTCCTGGAGGGACAGTCACA

CTCACCTGTGCTTCCAGTACTAGGCCTGTCACAACCAGTCACCATGCTAGCTGTTTCCAA

CAAAAGCCTTACCAAACACAACAGGGATTAAAATC-TACCACCAGCCTCCAGATCTCTGT

---------------TGTCCCTGCCCAATTCATAGGCTC------CTTGCTTGGAGACAA

GGCTGTCCTCACAATCACAGGGCCCCAGGGTGAAGATAAGACCACCTGTTACTAT-----

----

> VL6-69

;

;

TCCCAGGCTGTGGTGACTCAGGAATCTTCACTGTACATTTCTCCTGGAGGGACAGTCACA

CTCACCTGTGCTTCCAGTACTGGGGCTGTCACAACCAGTAACTATGCTGCCTGGGTCCAA

CAAAAACCCTCTGAAATACCAAAGGGGCTTATATA-TCATACCAGCACCCGAAACTCGGG

---------------AGTTCCTGCCCGATTCACAGGCTC------CTTACTTGGAGACAA

GGCTGCCTTCACCATTACAGGGGCCCAGACTGAAGATGAGGCCACTTATTACTGT-----

----

> VL6-68

;

;

TCCCAGGCTGTGGTGACTCAGGAATCTTCACTGTACATCTCTCCTGGAGGGACAGTCACG

CTCACCTGTGCTTCCAGTACTGGGGCTGTCACAACCAGTAACTACGTTGACTGGATCCAA

CAGAAACCATACCAAACACCCCAGCAGATAATTGG-TGACAACAGCAATCAGGTCCCCGG

---------------TGTCCCTGCCAGATTCTCAGGCTC------ATTGCTTGGAGACAA

GGCTGCCCTCACCATCACAGGGATCCAGCCTGAAGATGAGGCCACCTATTACTGT-----

----

> VL10-55

;

;

-----TTACACCCTCACTCAACCTCCCTCAGTGTCTGTGACTCCAGCACAGACAGCCAAA

ATCACATGCTCTGGAGAT----GACTTGGGGA-----ATAAGTATGCACACTGGTACCAG

CAGAAGCCAGGCCAGGCTCCTGTGCTGGTCATCTA-TGAAGATAGCAAGCGGCCTTCAAG

---------------GATTCCTGATCGATTCTCTGGATC------CAACTCAGGGAACGT

GGCCACCCTGACCATCACAGGGGTTCAAGCTGGGGATGAGGCTGTCTACTACTGT-----

----

> VL12-53

;

;

---TCCTATGTACTCACACAGCCACCCTCAGTGTCAGTGTCCCTGGGAGAGACAGTCACA

ATTACCTGCTCTGGAGAT----GCACTGCCAA-----AAAAATATGCATACTGGTTCCAG

CAGAAGCCAGGACAGACTCCTGTGCGAGTAATATA-CAAAGACAGCGAGCGGCCCTCAGG

---------------GGTCTCTGAGAGATTCTCTGGCTC------CAGCTCAGGGACCAC

AGCCACCCTGACCATCAGTGGAGTCCAGGCTGAAGATGAGGCTGATTATTACTGT-----

----

> VL12-52

;

;

---TCCTATGTACTCACACAGCCACCTTCAGTGTCGGTGTCCCCAGGAGAGACAGTCACA

ATTACCTGCTCTGGAAAT----GTACTACCAA-----AGAAATACGCACAATGGTTCCAG

CAGAAGCCAGGGAAAACT---GTGCAATTAATATA-CAAAAACAGTGAGTGGCCCTCAGG

---------------GGTCTCTGAGAGATTCTCTGGCTC------CAAGTCAGGGCCCAC

AGCCACCCTTACCATCAGCAGAGTCCAGGCTGAAGATGAGGCTGATTACTACTGT-----

----

> VL3-50

;

;

---TCCTATGTGCTGACGCAGCCATCTTCCATGTCAGTGTCTCTGGGAGAGACCATCAGC

CTGACATGTGATGGAAACA---ACATTGGAGG------TAGAAGAGTGCGCTGGTACCAG

CAGAAGCCACCCCAGTGCCCCATGCTGGTCATGTA-TAGTGATAACAACCGGCCCTCGGG

---------------GATTCCTGACCGGTTCTCCGGTGC------CAACTCAGGCAACCC

AGCCACACTGACCATCACAGGTGCCCAGGATGAGGACGAGGCCGACTATTACTGT-----

----

> VL3-48

;

;

---TCCTATGTGCTGACGCAGCCATCTTCCATGTCAGTGTCTCTGAAAGAGACAGTCAGG

CTGACCTGCGAGGGAAACA---ACATTGGAGA------TAAAGCTGTGCACTGGTACCAG

CAGAAGCCACCCCAGTCCCCCATGCTGGTCATGTA-TACTGATAAAAACCGACCCTCGGG

---------------GATTCCTGACCGGTTCTCTGGTGC------CAACTCGGGCAACAC

TGCCACACTGACCATCACTGGTGCCCAGGCTGAGGATGAGGCCGACTATTACTGT-----

----

> VL10-47

;

;

---TCTTACACCCTCACTCAACCTCCCTCAGTGTCTGTGACTCCAGCACAGACAGCCAAA

ATCACATGCTCTGGAGAT----GACTTGGGGA-----ATAATTATGCATCTTGGTACCAG

CAGAAGCCAGGCCAGGCTCCTGTGCTGGTCATCTA-TGAAGATAGCGAGCGGCCTTCAGC

---------------GATTCCTGATCGATTCTCTGGATC------CAACTCAGGAAATGT

GGCCACTCTGACAATCACAGGAGTACAAGCTGGAGATGAGGCTGTCTACTACTGT-----

----

> VL3-45

;

;

---TCCTATGTGCTGACGCAGCCATCTTCCATGTCAGTGTCTCTAAGAGAGACAGCCAGG

TTGACCTGTGAGGGAAACA---ACATTGGAAG------TAAATATGTACACTGGTACCAG

CAGAAGCCACTGCAGGCCCCCATGCTGGTCATG----AATAATAATAACCGGCCCTCTGG

---------------GATTCCTGACCGATTCTCCGGTGC------CAAGTCGGGCAACAT

GGCCACACTGACCATCACTGGCGCCCAGGCTGAGGACGAGGCCGACTATTACTGT-----

----

> VL11-43

;

;

---TCTTACATCTTGACACAGCCTCCCTCAGTGTCAGTGACCCCAGGGCAGACAGCCACA

ATCACCTGCTCCGGAGAT----AAACTCTCTA-----AACAATACGCACATTGGTACCAG

CAGAAGCCAGGCCAAGTGCCAACATTGCTCATCTA-TAAAGATAGTGAACGAGCATCAGG

---------------GATCCCTGACAGATTCTCTGGCTC------CAGCTCAGGAAACAC

AGCCACCTTGACCATCAGTGGGACCCAGGCTGCAGATGAGGCTGACTATTACTGT-----

----

> VL3-42

;

;

---TCCTATGTGCTGACGCAGCCATCTTCCATGTCAGTGTCTCTGAAAGAGACAGTCAGG

CTGACCTGCGAGGGAAACA---ACATTGGAGA------TAAAGCTGTGCACTGGTACCAG

CAGAAGCCACCCCAGTCCCCCATGCTGGTCATGTA-TACTGATAAAAACCGACCCTCGGG

---------------GATTCCTGACCGGTTCTCTGGTGC------CAACTCGGGCAACAC

TGCCACACTGACCATCACTGGTGCCCAGGCTGAGGATGAGGCCGACTATTACTGT-----

----

> VL10-41

;

;

---TCTTACACCCTCACTCAACCTCCCTCAGTGTCTGTGACTCCAGCACAGACAGCCAAA

ATCACATGCTCTGGAGAT----GACTTGGGGA-----ATAAGTATGCACACTGGTACCAG

CAGAAGCCAGGCCAGGCTCCTGTGCTGGTCATCTA-TGAAGATAGCGAGCGGCCTTCAGG

---------------GATTCCTGATCGATTCTCTGGATC------CAACTCAGGAAATGT

GGCCACCCTGACCATCACAGGGGTACAAGCTGGAGATGAGGCTGTCTACTACTGT-----

----

> VL3-39

;

;

---TCCTATGTGCTGACACAGCCGTCTTCCATGTCAGTGTCTCTGGGAGAGACAGCCAGG

CTGACCTGTGAGGGAAACA---ACATTGGAGG------TAAATATGTGTACTGGTACCAG

CAGAAGCCACCCAAGGCCCCCTTGCAGGTCATGTA-TAAGAATAACAACCGGCCATCTGG

---------------TATTCCCGACCGGTTCTCCGGTGC------CAAGTCGGGCAACAT

GGCTACACTGACCATCACTGGCGACCAGGCTGAGGATGAGGCCGACTATTACTGT-----

----

> VL11-35

;

;

---TCTTACATCTTGACACAGCCTCCCTCAGTGTCAGTGTCCCCAGGGCAAACAGCCGCA

ATCACCTGCTCCGGAGAT----AAACTCTCCA-----AACGATATGCATATTGGTACCAG

CAGAAGCCAGGCCAAGCTCCAGCATTGCTCATCTA-TGAAGATAGTAAACGACCATCAGG

---------------GATCCCTGACAGATTCTCTGGCTC------CAGCTCAGGAAACAC

AGCCACCTTGACCATCAGTGGGACCCAGGCTGCAGATGAGGCTGACTATTACTGT-----

----

> VL3-34

;

;

---TCCTATGTGCTGACGCAGCCATCTTCCATGTCAGTGTCTCTAAGAGAGACAGCCAGG

CTGACCTGTGAGGGAAACA---ACATTGGAGG------TAAATATGTACACTGGTACCAG

CAGAAGCCACTGCAGGCCCCCATGCTGGTCATTTA-TGATAACAACAACTGGCCCTCGGG

---------------GATTTCTGACCGGTTCTCTGGTGC------CACGTCGGGCAACAC

GGCCACACTGACCATCACTGGCGCCCAGGCTGAGGACGAGGCCGACTATTACTGT-----

----

> VL3-32

;

;

---TCCTATGTGCTGACGCAGCCATCTTCCATGTCAGTGTCTCTAGGAGAGACAGCCAGC

CTGACCTGTGAGGGAGACA---ACATTGGAAT------TTATTATGTGTACTGGTACCAG

CAGAAGCCACCCAAGGCCCCCATGCTGGTCATGTA-TAGTAATAACAACCGGCCCCCGGG

---------------GATTCCTGACAGCTTCTCCAGTGA------CAACTGGGGCAACAC

GGCCACACTGACCATCACTGGTGTCCAGGCTGAGGACGAGGCTGACTATTACTGT-----

----

> VL10-31

;

;

---TCTTACACCCTCACTCAACCTCCCTCAGTGTCTGTGACTCCAGCACAGACAGCCAAA

ATCACATGCTCTGGAGAT----GACTTGGGGA-----ATAAGTATGCATACTGGTACCAG

CAGAAGCCAGGCCAGGCTCCTGTGCTGGTCATCTA-TGAAGATAGCGAGCGGCCTTCAGG

---------------GATTCCTGATCGATTCACTGGATC------CAACTCAGGGAACGT

GGCCACCCTGACAATCACAGGGGTACAAGCTGGGGATGAGGCTGTCTACTACTGT-----

----

> VL12-30

;

;

---TCCTATGTGCTCAAACAGCCACCTTCAGTGTCGGTGTCCCTGGGACAGACAGCCTCA

GTTACCTGCTCTGGAAAC----GAACTGCCAA-----GTAGATATGCATGTTGGTTCCAG

CAGAAGCCAGGGCAGCCTCCTGTGCAAGTAATATA-CAAAGACAGTGAGTGGTCCTCAGG

---------------AATCTCTGAGAGATTGTCTGGTTC------CAACTCAGGGACCAC

AGCCACCCTGACCATCAACGGAGTCCAGGCTGAAGATGAGGCTGATTATTACTGT-----

----

> VL12-29

;

;

---TCCTATGTACTCACACAGCCACCTTCAGTGTCAGTGTCCCCGGGACAGACAGCCACA

ATTACCTGCTCTGGAGAA----GCACTGCCTA-----AAAGATACGCACAGTGGTTCCAG

CAGAAGCCAGGACAGACTCCTATGAGCGTAATATA-CAAAGACAGTGAGCGGCCCTCAGG

---------------GATCTCTGACAGATTCTCTGGCTC------CAGCTCAGGGACCAC

AGCCACCCTGACCATTAGCGGAGTTCAAGCTGGAGATGAGGCTGATTACTACTGT-----

----

> VL3-27

;

;

---TCCTATGTACTGACACAGCCATCTTCCATGTCAGTGTCTCCTGGAGAGACAGCCAAG

CTGACCTGTGAGGGAAACA---ACATTGGAAG------AAAAAGTGTGCAATGGTTCCAG

CAGAAGCCACCCCAGGCCCCCATGCTGGTCATATA-TGCTGATAACCTACGGCCCTCCAA

---------------GATTCCCAACCGATTCTCCGGTGC------CAACTCGGGCAACAC

GGCCACACTGACCATCACGGGCGCCAAGGCTGAGGATGAGGCCGACTATTACTGT-----

----

> VL12-24

;

;

---TCCTATGTACTCACACAGTCACCTTCAGTGTCAGTGTCCCCGGGACAGACAGCCACA

ATTACCTGCTCTGGAGAT----GAACTGCCAA-----GTACATTTGCACACTGGTACCAG

AAGAAACCAGGACAGACTCCTGTGAACATAATACA-GAAAGACAGTGAGCGGCCCTCAGG

---------------GGTCTCTGAGAGATTCTCTGGCTC------CAGCTCAGGGACCAC

AGCCACCCTGACCATCAGCAGAGTCCAAGCTGAAGATGAGGCTGATTACTACTCT-----

----

> VL3-21

;

;

---TCGTATGTGCTGACGCAGCCATCTTCCATGTCAGTGTCTCTGGGAGAGACCGCCATC

CTGACATGTGATGGAAACA---ACATTGGAGG------TAGAAGAGTGCACTGGTACCAG

CAGAAGCCACCCCAGACCCCCATGCTGGTCATATA-TGCTGATAACAACCGGCCCTCTGA

---------------GATTCCTAATCGGTTCTCCGGTGC------CAAGTCGGGAAACAC

GGCCACCCTGACCATCACTGGCGCCCAGGCTGAGGATGAGGCCGACTATTACTGT-----

----

> VL11-19

;

;

---TCTTATATCTTGACACAGCCACCCTCAGTGTCAGTGTCCCCAGGGCAGACAGCTACA

ATCACCTGCTCTGGAGAG----AAACTGTCTG-----AACGGTATGCATATTGGTACCAG

CAGAAACCAAGTCAGGCTCCTGCCCTGGTCATCTA-TAATGATAGCGAACGGCCCTCAGA

---------------GATCCCTGACAGATTCTCTGGCTC------CAGCTCTGGAAACAC

AGCCACTTTGACCATCAGTGGGGCCCAGGCTGCCGATGAGGCTGATTATTACTGT-----

----

> VL3-16

;

;

---TCCTATGTGCTGACGCAGCCATCTTCCATGTCAGTGTCTCTGGGAGAGACAGTCAGG

CTGACCTGTGAGGGAAACA---ACATCGGAGG------TAAAAATGTGTACTGGTATCAG

CAGAAGCCACCCCAGGCCCCTATGCTGGTCATGTA-CAGTGATAATACTCGGCCCTCTGG

---------------GATTCCTGACCGGTTCTCCGGTGC------CAAATCTGGCAACAC

GGCCACACTGACCATCACTGGCACCCAGGATGAGGATGATGCTGACTATTACTGT-----

----

> VL3-13

;

;

---TCCTATGTGCTGACGCAGCCATCTTCCATGTCAGTGTCTCTTGGAGGGACAGTCAGG

CTGACCTGCGAGGGAAACA---ACATTGGAGG------TAAAGCTGTGCAGTGGTACCAG

CAGAAGCCACCCCAGGCCCCCATGCTGGTCATGTA-TAATGGTAACAGCCGGCCCTCAGA

---------------GATTCCGGATCGATTCTCTGGTGC------CAACTCGGGCAACAC

GGCCACACTGACCATCTCTGGTGCCCAGGATGAGGATGAGGCCAGCTATTACTGT-----

----

> VL3-10

;

;

---TCCTATGTGCTGACGCAGCCATCTTCCATGTCAGTGTCTCTGAAAAAGACAGTCAGG

CTGACCTGCGAGGAAAACA---ACATTGGAGA------TAAAGCTGTGCACTGGTACCAG

CAGAAACCACCCCAGGCCCCCATGCTGGTCATGTA-TATTGATAACAACCGACCCTCGGG

---------------GATTCCTGACCGGTTCTCTGGTGC------CAACTCAGGCAACAC

CGCCACACTGACCATCACTGGCGCCCAGGCTGAGGACGAGGCCGACTATTACTGT-----

----

> VL10-9

;

;

---TCTTACACCCTCACTCAACCTCCCTCAGTGTCTGTGACCCCAGCACAGACAGCCAAA

ATCACATGTTCTGGAGAT----AACTTGGGGA-----ATAAGTATGCATACTGGTACCAG

CAGAAGCCAGGCCAGGCTCCTGTGCAGGTCATCTA-TGAAGATAGCGAGCGGCCTTCAGG

---------------GATTCCTGATCGATTCACTGGATC------CAACTCAGGGAACGT

GGCCACCCTGACAATCACAGGGGTACAAGCTGGGGATGAGGCTGTCTACTACTGT-----

----
