# Supplementary figures and images for: Immunoglobulin Genomics in the Guinea Pig (Cavia porcellus)
Source: PLoS One. 2012 Jun 22;7(6):e39298. doi: 10.1371/journal.pone.0039298 (PMC3382241; doi:10.1371/journal.pone.0039298)

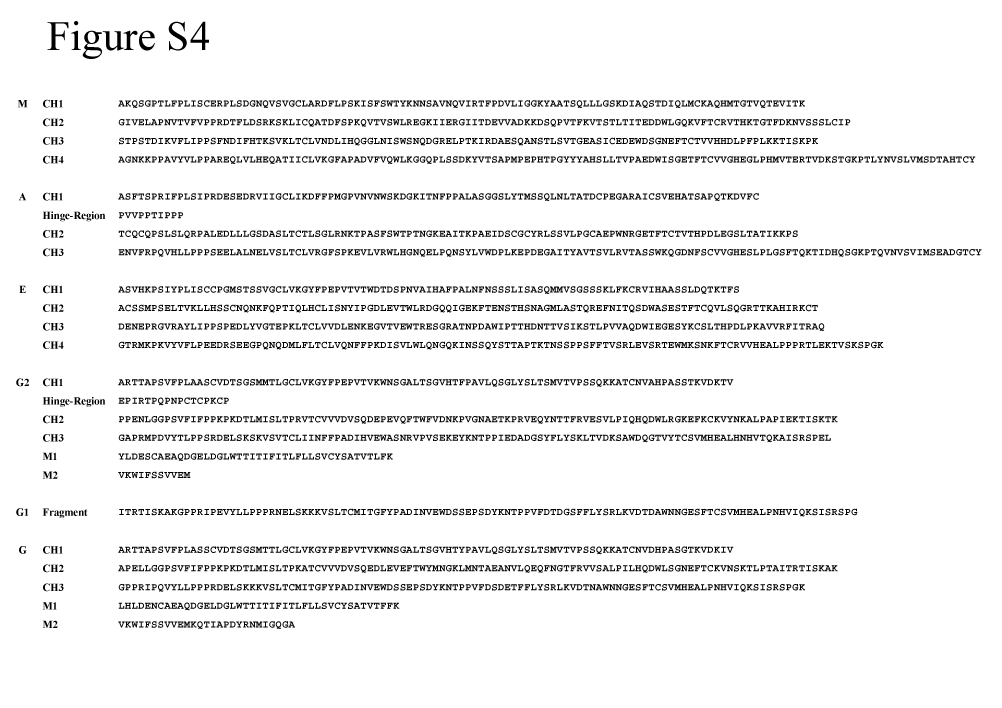

Supplement: Figure S4 — Guinea pig immunoglobulin heavy chain constant region encoding gene amino acid sequences. Four guinea pig encoding gene amino acid sequences of constant region (IgM, IgG, IgE and IgA) are cloned by 3′RACE. (TIF) [file pone.0039298.s004.tif]

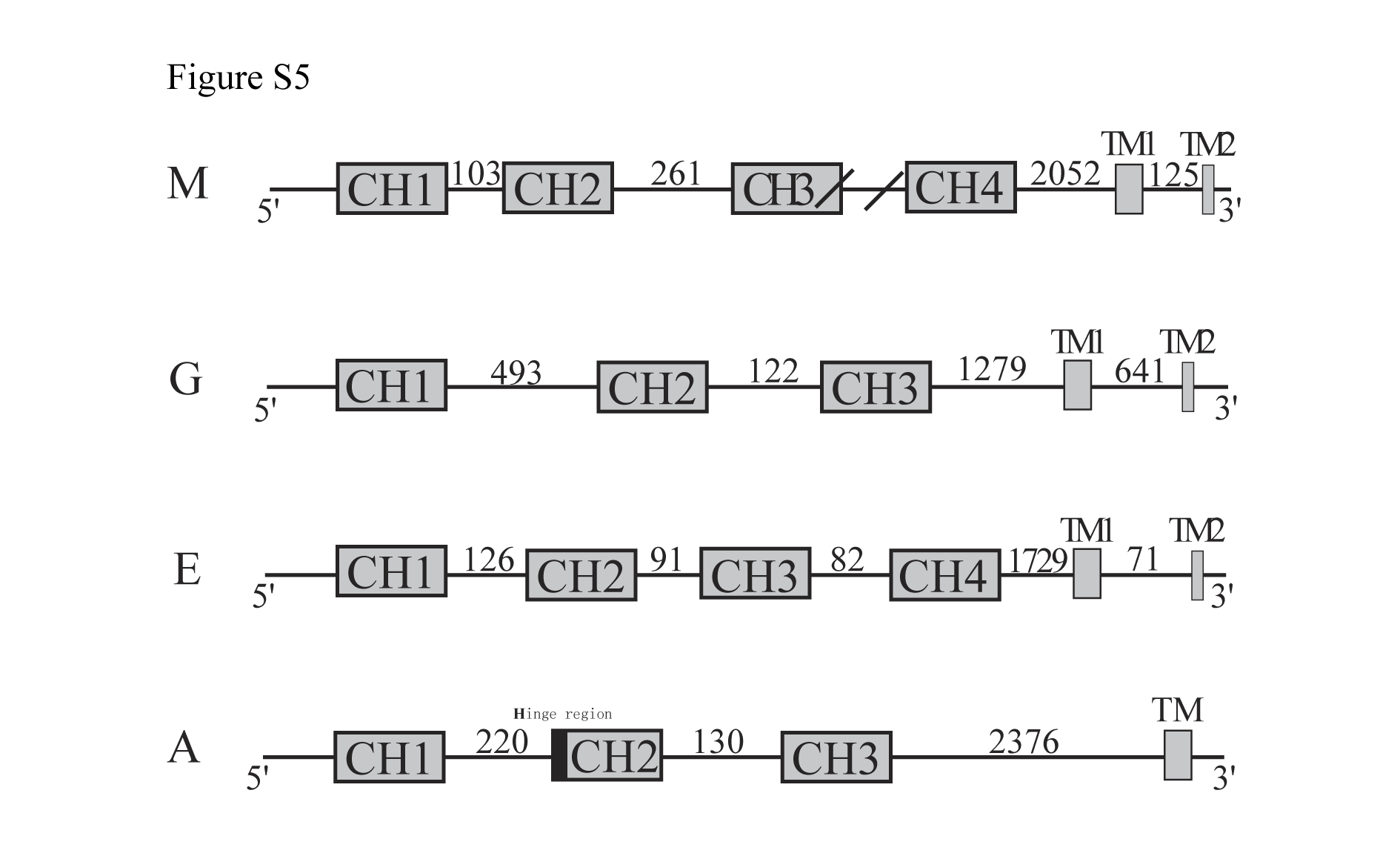

Supplement: Figure S5 — Analysis results of the guinea pig Ig μ, γ, ε and α genes in scaffold 54. Four guinea pig constant region encoding genes of tansmembrane type for IgM, IgG, IgE and IgA were identified by bioinformatics analysis. (TIF) [file pone.0039298.s005.tif]

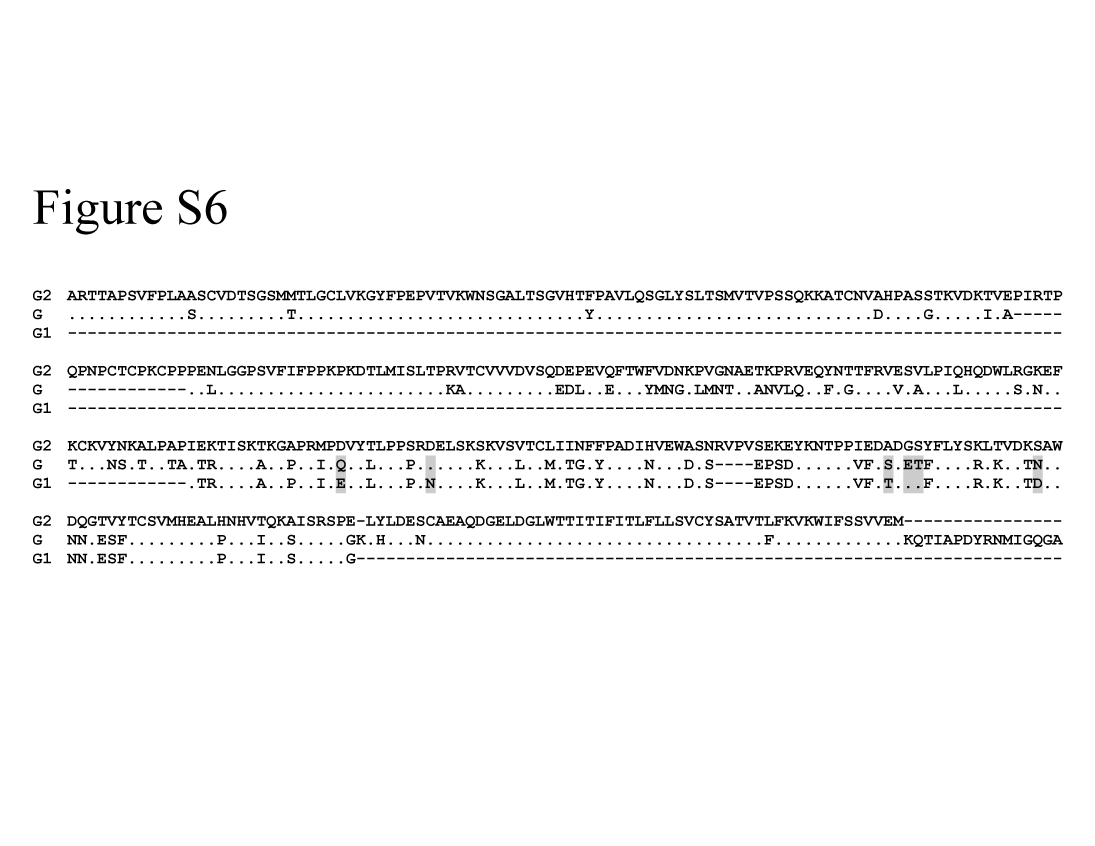

Supplement: Figure S6 — Alignment of the IgG amino acid sequences of the guinea pig. Alignment of IgG1, IgG2 and IgG squence of guinea pig. Dots indicate similar residues as in G1 and G, whereas dashes indicate gaps introduced for optimal alignment. The diference is represented by grey background between G and G1. (TIF) [file pone.0039298.s006.tif]

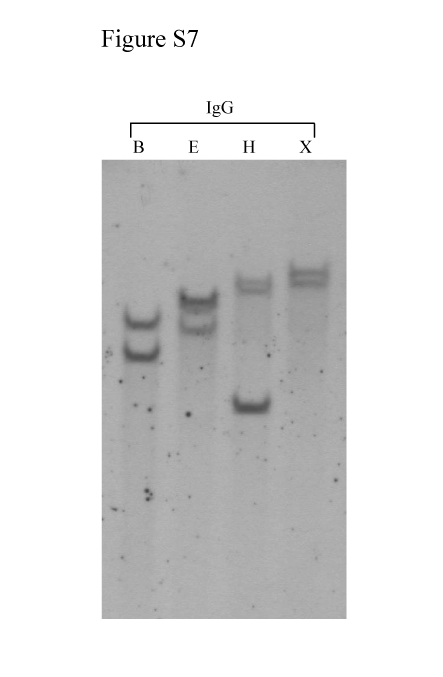

Supplement: Figure S7 — Southern blotting analysis of guinea pig genomic DNA. Southern blotting analysis of guinea pig heavy chain constant region IgG genes. The genomic DNA was digested with BamH I (B), EcoR I (E), Hind III (H) and Xba I (X), and hybridized with probes for IgG-CH1 sequence. (TIF) [file pone.0039298.s007.tif]
