# Supplementary material for: Immunoglobulin Genomics in the Guinea Pig (Cavia porcellus)
Source: PLoS One. 2012 Jun 22;7(6):e39298. doi: 10.1371/journal.pone.0039298 (PMC3382241; doi:10.1371/journal.pone.0039298)
Supplement: Table S1 — Primers were designed for amplify guinea pig four classes immunoglobulin M, E, A, and G genes. (DOC) [file pone.0039298.s009.doc]

**Table S1**

| 3' RACE-Reverse transcription primer | AACTGGAAGAATTCGCGGCCGCAGGAATTTTTTTTTTTTTTTTTT |
| --- | --- |
| G- sense primer | GCCAGAACAACAGCCCCTT |
| A- sense primer | CCTCGCATCTTCCCACTGA |
| E- sense primer | GTCCTGTCTTACAGCTTCTG |
| M- sense primer | CCCAACTCTCTTTCCCCTC |
| 3' RACE- antisense primer1 | AACTGGAAGAATTCGCGGC |
| 3' RACE- antisense primer 2 | AAGAATTCGCGGCCGCAGGAA |
| VH1-95- sense primer | TGGCCTGGTAAAGCCCT |
| VH1-95- antisense primer | CAGTAATAGGTGGCTGTGTCTTC |
| VH2-17- sense primer | TGCAGCTGCAGGAGTCAGGA |
| VH2-17-antisense primer | CCTTGCACAGTAATACATGGCC |
| VH3-157- sense primer | GAGGAGCAACTGGTGGAGTC |
| VH3-157-antisense primer | GTCTCAGGCTGCTCATCTGCAG |
| IgG-CH1- sense primer | GAACAACAGCCCCTTCCGTCTT |
| IgG-CH1- antisense primer | CAATCTTGTCCACTTTGGTGCC |
